# Exploring the potential of polyphenols in major depression: from molecular function to clinical therapy

**DOI:** 10.3389/fnut.2026.1725745

**Published:** 2026-05-18

**Authors:** Shiyu Zhu, Qinming Yu, Yuhan Jiang, Qiuyue Zu, Wendan Pang

**Affiliations:** 1 Sports and Military Training Department, Zhejiang A&F University, Hangzhou, China; 2 School of Humanities and Management, Heilongjiang University of Chinese Medicine, Harbin, Heilongjiang, China; 3 Department of Rehabilitation, Tiantai People's Hospital of Zhejiang Province (Tiantai Branch of Zhejiang Provincial People's Hospital), Taizhou, Zhejiang, China

**Keywords:** antidepressant effects, dietary therapy, major depressive disorder, neurological disorders, polyphenol

## Abstract

Major depressive disorder (MDD) is a highly prevalent and debilitating psychiatric condition and remains a major contributor to global disease burden. Recent global burden of disease analyses indicate that both the incidence and prevalence of MDD have increased over the past decades, with projections suggesting continued growth worldwide. These trends highlight an urgent need to explore novel and complementary therapeutic strategies. MDD is a complex mental health condition characterized by disturbances in mood, cognition, and behavior, and its pathophysiology involves multiple biological systems. During depressive episodes, inflammatory pathways are activated, mitochondrial function is impaired, oxidative stress markers are increased, and antioxidant capacity is diminished. Approximately 30% of individuals with depression do not respond adequately to conventional pharmacological treatments. Consequently, increasing attention has been directed toward the impact of bioactive food components on neurological disorders, including MDD. Polyphenols, a class of bioactive compounds abundantly found in plant-based foods and beverages, have garnered significant interest for their potential role in mitigating MDD. While numerous animal studies suggest antidepressant-like effects of polyphenols, particularly catechins, curcumin, resveratrol, and quercetin, clinical research remains limited, with randomized controlled trials primarily focusing on curcumin and anthocyanins. In this paper, evidence from preclinical and clinical studies is reviewed to explain how polyphenols may act as antidepressant agents. Their effects are described through actions on neurotransmitter systems, reductions in inflammation and oxidative stress, and support of neuroplasticity. In conclusion, current findings indicate that polyphenols may provide meaningful benefits in managing MDD due to their wide-ranging biological effects. Integrating polyphenols into clinical practice therefore represents a promising approach for improving treatment outcomes. Future directions include expanding clinical trials to evaluate a broader spectrum of polyphenols, developing strategies to improve bioavailability, standardizing product formulations, examining potential interactions with conventional medications, and promoting public education on dietary strategies that support mental health.

## 1 Introduction

Major depressive disorder (MDD) is a prevalent psychiatric condition impacting approximately 185 million individuals worldwide (1). MDD is the second largest contributor to chronic disease burden, measured by “years lived with disability” (2). Furthermore, MDD is linked to a higher risk of developing other conditions like diabetes, heart disease, and stroke, which add to its overall impact on health (3). Moreover, MDD can result in death by suicide. It is estimated that up to half of the 800,000 annual suicides globally occur during a depressive episode. Individuals with MDD are nearly 20 times more likely to die by suicide compared to the general population (4). Neuroimaging studies have identified key pathological features of MDD, including reduced structural volumes in both cortical and subcortical areas, decreased gray matter volume, and significant alterations in the size and density of microglial cells (5, 6). These changes are also associated with abnormal neurotransmitter regulation and disruptions in gut microbiota (7). While the exact cause of MDD is not fully understood, various hypotheses have been suggested. These include the monoamine theory, the role of neurotrophins, the hypothalamic-pituitary-adrenal (HPA) axis, neuroinflammation, oxidative stress, and the microbial-gut-brain (MGB) axis (8). Standard treatments for depression often include antidepressant medications like selective serotonin reuptake inhibitors (SSRIs) and/or psychotherapy, such as cognitive behavioral therapy (CBT). In cases of severe or treatment-resistant MDD, other biological therapies like electroconvulsive therapy (ECT) may be utilized (9). Several new and emerging treatments are currently being tested in clinical trials or have recently been adopted into clinical practice. These include biological interventions like transcranial magnetic stimulation (TMS), ketamine, and psychedelics, as well as lifestyle interventions such as dietary changes and exercise (10, 11). Patients with MDD often struggle with incomplete recovery, medication side effects, withdrawal symptoms, and relapse. These issues pose serious risks to their lives and wellbeing and also have a substantial negative impact on society (12).

The complexities surrounding treatment resistance in MDD involve a range of factors, including diverse mechanisms. The effectiveness of antidepressant (AD) treatment faces obstacles due to the lack of a dependable biomarker for MDD, leading to reliance on clinical evaluation using standardized rating scales (13). Despite progress in pharmacological interventions that target monoamine function, such as serotonin, noradrenaline, dopamine, and melatonin, many patients still struggle to attain remission (14). Response to treatment is categorized into non-response, partial response, response, and remission; however, research indicates that only a small portion of patients achieve full remission even after undergoing multiple trials of AD drugs (15). Addressing MDD that does not respond to typical treatments poses notable hurdles. A key challenge stems from the intrinsic constraints of the treatment-resistant depression (TRD) concept. The notion of TRD has arisen to characterize instances where patients do not respond sufficiently to treatment. Typically, this is defined as a lack of response to two or more appropriate trials of AD drugs from different classes (16). While TRD serves as a valuable tool in clinical and research domains, it fails to fully encompass the diverse clinical presentations observed in practice. Defining response and treatment adequacy can be challenging due to unclear boundaries, including cases of partial response and full refractoriness. Moreover, the complexity is heightened by varying symptom presentations, the alignment of treatments with specific symptoms, and the influence of comorbidities and psychosocial factors (17). Furthermore, translational research utilizing animal models sheds light on diverse mechanisms underlying AD non-response, notably stress-induced behavioral impairments. Potential biomarkers, encompassing genetic examinations, plasma assessments, and brain imaging, exhibit encouraging prospects in anticipating treatment outcomes (18). In clinical practice, practical implications such as augmentation therapy involving thyroid hormones have become commonplace, whereas novel strategies like therapeutic drug monitoring and alternative augmentation methods are currently being explored (19). The considerable diversity observed among patients identified as TRD presents a substantial challenge in enhancing care strategies. This diversity originates from the lack of specificity in diagnostic criteria, emphasizing the necessity for a systematic integration of biomarkers to precisely characterize the clinical phenotypes of MDD. Despite the obstacles encountered, investigating TRD and the lack of response to treatment remains pivotal for enhancing our comprehension of MDD's underlying mechanisms and enhancing patient wellbeing (20).

MDD presents with a complex etiology and is clinically manifested by symptoms such as anhedonia (loss of pleasure), feelings of hopelessness, suicidal thoughts and behaviors, cognitive deficits, and persistent low mood (dysthymia) (21, 22). There are currently numerous treatments for MDD, each addressing different underlying causes. Recently, connections between nutrition and mental health have been identified. Diets or supplements rich in antioxidants and anti-inflammatory properties may offer therapeutic benefits. Evidence suggests that the quality of individuals' diets may impact the likelihood of developing MDD (23). Recent epidemiological studies suggest that diets rich in fruits, vegetables, whole grains, fish, olive oil, low-fat dairy, and antioxidants, while low in animal products, have been linked to a reduced risk of MDD (24). A common feature of these food groups is their natural abundance of polyphenols, which are compounds known for their antioxidant and anti-inflammatory properties. Consuming dietary polyphenols has been linked to various health benefits, including lower risks of mortality, cardiovascular diseases, and several types of cancer (25). Findings from *in vitro* and *in vivo* research indicate that various dietary polyphenols, including flavonoids, phenolic acids, and lignans, may contribute to brain health, particularly in relation to depression (26). Dietary polyphenols have demonstrated several mechanisms potentially involved in the pathophysiology of depression, such as anti-neuroinflammatory effects, inhibition of neuronal apoptosis, and promotion of adult neurogenesis (27).

Although substantial progress has been made in understanding the biological underpinnings of MDD and the challenges associated with treatment resistance (14, 17), important gaps remain in how nutritional strategies, particularly polyphenol-rich foods and supplements, can be integrated into depression management. Existing reviews often address only isolated mechanisms, such as inflammation (28), oxidative stress and neuroprotection (29), or gut–brain interactions (30), while others focus exclusively on preclinical evidence or specific subclasses of polyphenols (31, 32). As a result, the current literature lacks a consolidated evaluation that integrates both preclinical and clinical findings to provide a comprehensive perspective on the therapeutic potential of polyphenols in MDD. Furthermore, considerable variability across studies in polyphenol types, dosages, formulations, and delivery methods has limited the translation of existing evidence into clear clinical recommendations (33, 34). In light of these issues, a comprehensive evaluation that unifies mechanistic and clinical insights is needed.

The objective of this review is to explore the multifaceted nature of treatment challenges in MDD and to examine how polyphenol-rich dietary factors may contribute to improved therapeutic outcomes. By bringing together evidence from animal and human studies, this review aims to clarify the major biological pathways through which polyphenols might influence depressive symptoms and complement existing treatments. Additionally, it seeks to highlight the limitations in current research and outline future directions that may enhance diagnostic precision and support more personalized nutritional and therapeutic strategies for individuals with MDD.

## 2 Pathophysiology of major depressive disorder

**Figure 1:**
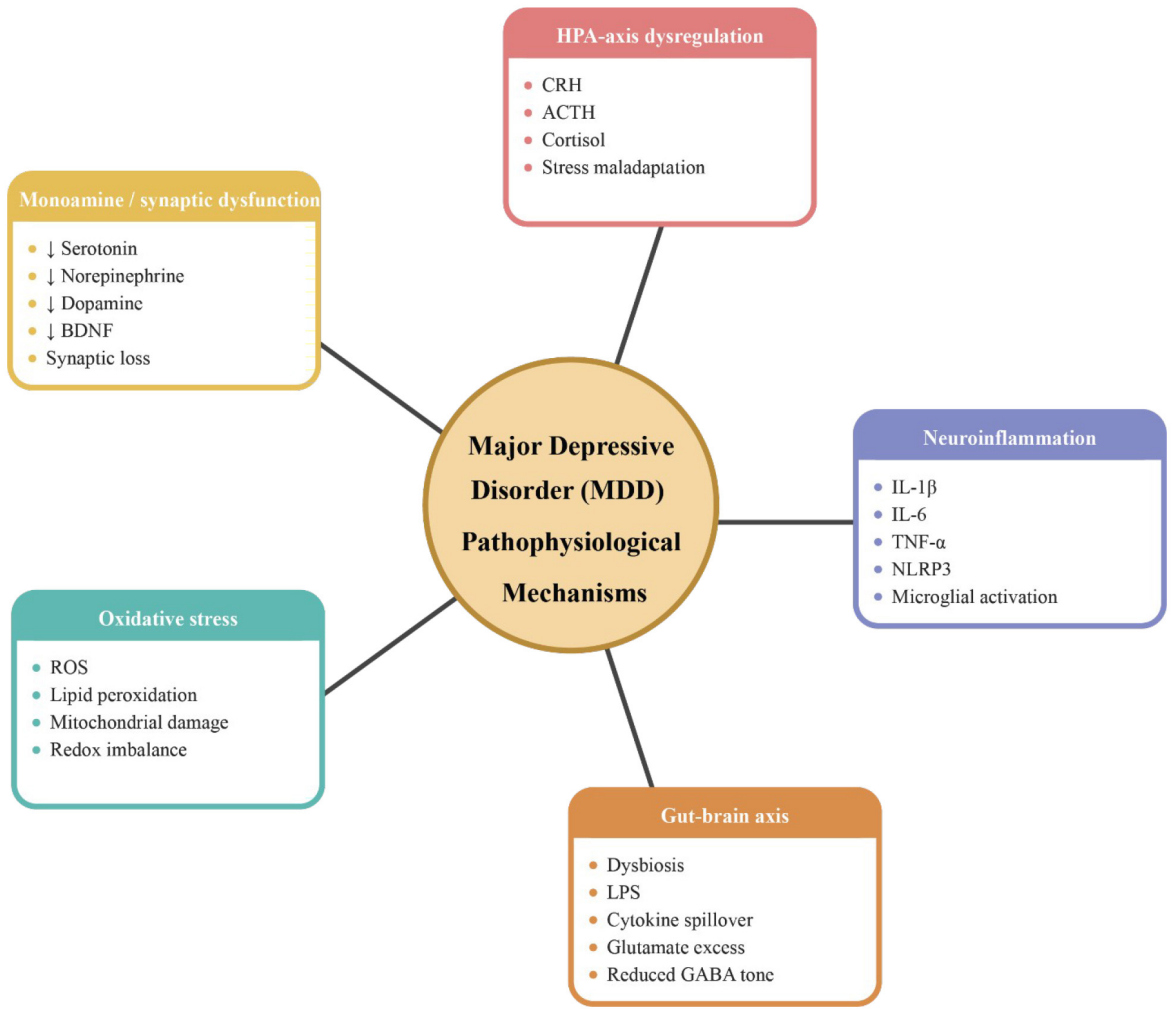
An illustration of major depressive disorder pathophysiology.

MDD is a complex psychiatric condition with a multifactorial pathophysiology. It involves intricate interactions between genetic, neurobiological, and environmental factors, leading to alterations in brain structure and function. Key aspects of the pathophysiology of MDD include neurotransmitter dysregulation, neuroendocrine abnormalities, neuroinflammation, and impaired neuroplasticity (Figure 1).

### 2.1 Monoamine theory

The monoamine hypothesis, developed from the unexpected discovery of antidepressants (ADs) in the 1950s, proposes that changes in mood in MDD are caused by a reduction in neurotransmitters in neuronal synapses (35). This theory has been crucial for creating ADs that boost the levels of serotonin (5-HT), norepinephrine, and dopamine in the brain (36). Although widely prescribed, these medications fully benefit only about one-third of patients, and lower doses are often used to reduce side effects. The exact mechanisms of action, particularly for selective serotonin reuptake inhibitors (SSRIs), are not well understood, and long-term use is typically necessary for effective treatment (37). Studies suggest that prolonged use of ADs results in the desensitization or downregulation of 5-HT receptors, including 5-HT1A receptors. Fluoxetine, a widely used SSRI, enhances 5-HT synaptic transmission by desensitizing terminal 5-HT inhibitory autoreceptors (38). ADs are believed to influence gene expression and stimulate neuroplasticity, thereby increasing their effectiveness with prolonged use. For example, fluoxetine has been observed to cause enduring neuroplastic changes in the medial prefrontal cortex (mPFC), which result in increased expression of brain-derived neurotrophic factor (BDNF) and associated proteins (39). The malfunctioning of monoamine oxidase (MAO) enzymes, responsible for breaking down neurotransmitters, significantly contributes to MDD (40). Increased MAO activity reduces neurotransmitter levels in the synapse, hindering synaptic functionality (40, 41). MAO inhibitors, particularly those that target MAO-A, have been effective in treating MDD by inhibiting the breakdown of essential neurotransmitters. The degradation of neurotransmitters by MAO enzymes generates neurotoxic by-products, such as hydrogen peroxide, which can form reactive oxygen species (ROS) and lead to neuronal apoptosis through oxidative stress (42, 43).

Although the monoamine hypothesis has been prominent in the study and treatment of MDD, recent perspectives indicate its limitations in capturing the complexity of the disorder. Despite this, the hypothesis has served as a useful guide for comprehending mood disorders and has stimulated the identification of novel treatment avenues and therapeutic approaches (44). Present investigations extend beyond monoamines, encompassing wider neurobiological and genetic elements implicated in the pathophysiology of MDD.

### 2.2 Glutamate and γ-aminobutyric acid (GABA)

Research indicates that glutamate and GABA neurotransmitters are associated with MDD, demonstrating reduced levels in patients, notably in the mPFC and amygdala regions (45). Astrocytes are vital in modulating glutamate levels by absorbing them through excitatory amino acid transporters (EAATs) and converting them into glutamine (46). Prolonged stress may decrease the number of astrocytes, resulting in the buildup of glutamate and the risk of excitotoxicity (47). Ketamine, recognized as a fast-acting antidepressant, functions by blocking N-methyl-d-aspartate (NMDA) receptors, thus triggering the release of glutamate (48). Two hypotheses surround the mechanism of ketamine; one suggests it involves disinhibition, while the other proposes a direct interaction with the NMDA receptor. Additionally, AMPA (α-amino-3-hydroxy-5-methyl-4-isoxazolepropionic acid) receptors play a role, activating the mTOR (mammalian target of rapamycin) signaling pathway, which could potentially counteract deficits in synaptic proteins. Exploration into the actions of ketamine beyond the NMDA receptor is ongoing.

### 2.3 Neuroinflammation

Recent findings supporting the neuroimmune hypothesis indicate a connection between impaired central and peripheral immune functions and neurobiological alterations in MDD (49). Cases of antidepressant-resistant MDD suggest that abnormal inflammatory and immune processes play significant roles (50). Persistent overstimulation of immune cells, including microglia, monocytes, and macrophages, adversely impacts neurobiological structure and function in MDD (51). Proinflammatory mediators, such as cytokines IL-1 (interleukin-1), IL-6 (interleukin-6), and TNF-α (tumor necrosis factor-α), are found in elevated levels in the brains and peripheral tissues of many MDD patients (52). These cytokines activate the NF-κB pathway, leading to the upregulation of inflammatory genes like COX-2, which in turn produces prostaglandins and exacerbates inflammation (53). Microglia and astrocytes are key regulators of the proinflammatory and anti-inflammatory balance in the central nervous system (CNS) (54). Peripheral cytokines can penetrate the blood-brain barrier (BBB), raising proinflammatory cytokine levels further (55). When astrocytes are activated, they produce IL-6, which affects both neuroprotection and neurodegeneration (56). Astrocytes maintain glutamate balance, and even minor disruptions can adversely affect synaptic neuroplasticity (57). In rats, administering bacterial lipopolysaccharide (LPS) triggers cytokine production in the CNS, primarily from monocytes and microglia (58). IL-1β may activate the Nod-like receptor pyrin containing 3 (NLRP3) inflammasome, a significant target for MDD therapy. Elevated IL-1β levels in the CNS are associated with MDD symptoms such as lethargy, sleep disturbances, and appetite changes (59). TNF-α and IL-1 amplify inflammation by activating peripheral monocytes and macrophages, resulting in elevated serum IL-8 levels that draw more leukocytes to inflammation sites (60). Psychological stress prompts interaction between the brain and immune system, causing monocytes to traverse the BBB and release proinflammatory cytokines through the NF-κB pathway, impacting neurons, astrocytes, and microglia. This leads to the retraction of neuronal dendrites, reduced neurogenesis in the hippocampus, and the loss of dendritic spines in the prefrontal cortex. Consequently, depressive symptoms such as headaches, nausea, fatigue, anhedonia, and social withdrawal manifest (61, 62).

### 2.4 Gut-brain axis

The role of microbiota in causing generalized inflammation is widely acknowledged. Disturbances in gut microbiota caused by antibiotics elevate the risk of depression by 20–50% (63). Lower alpha diversity is linked to increased depressive symptoms, while beta diversity is associated with MDD and other mental health conditions (64). Depression is associated with reduced levels of *Firmicutes* and fewer anti-inflammatory, butyrate-producing bacteria such as *Faecalibacterium* and *Coprococcus*, while showing increased levels of pro-inflammatory bacteria like *Eggerthella* (65, 66). Stool samples from depressed individuals exhibit higher levels of gram-negative bacteria such as *Bilophila* and *Alistipes*, and lower levels of *Anaerostipes* and *Dialister*, compared to those from healthy individuals (67). Dysfunction of the gut barrier permits bacterial migration, which activates the immune system and results in neuroinflammation. This condition correlates with the severity of depression, characterized by increased levels of IgA and IgM against bacterial LPS and elevated pro-inflammatory cytokines (IL-6, IL-1β, TNF-α) (68, 69). Stress negatively impacts the diversity of gut flora, while psychobiotics (probiotics and prebiotics) can generate neurotransmitters and short-chain fatty acids (SCFAs) that affect the nervous system. SCFAs influence the functions of neurons, microglia, and astrocytes, and can impact BBB, potentially causing neurodegeneration (70–72).

### 2.5 Endocrine dysregulation

Endocrine system irregularities, such as altered growth hormone (GH) levels, HPA axis dysfunction, and thyroid abnormalities, play a crucial role in MDD (73). Individuals with depression exhibit impaired GH release and a reduced GH response to clonidine and apomorphine (74). The thyroid gland generates tri-iodothyronine (T3) and tetra-iodothyronine (T4) hormones, controlled by thyroid-stimulating hormone (TSH), and dysfunction in thyroid activity is associated with depression (75). Imbalances in thyroid hormones can lead to symptoms such as sleep disturbances, weight loss, and psychomotor retardation in individuals with MDD (76). Furthermore, thyroid hormones might function as co-transmitters in the adrenergic nervous system (77). The HPA axis plays a vital role in the development of MDD, with issues such as defects in glucocorticoid mechanisms, overproduction of cortisol and corticotrophin-releasing factor (CRF), and dysfunctional corticosteroid receptor signaling (78). CRF controls HPA activity, which boosts adrenocorticotrophic hormone (ACTH) and cortisol production (79). In individuals with depression, elevated CRF levels lead to increased cortisol, causing severe symptoms like hopelessness, weight loss, sleep disturbances, psychomotor retardation, and heightened reactions to stress (80).

Long-term administration of corticosterone (CORT) in rodents reduces BDNF levels in the hippocampal dentate gyrus while increasing proBDNF, which may contribute to depression. While short-term stress activates the HPA axis temporarily, permitting a return to homeostasis, prolonged HPA activation and high glucocorticoid levels result in brain alterations associated with depression (81, 82). Increased levels of hydroxycorticosteroids, a derivative of cortisol, are linked to suicidal tendencies in individuals with depression (83). Glucocorticoid receptors (GR), which play a role in regulating stress responses, show reduced expression in the hippocampus under chronic stress. FKBP51, produced by the FKBP5 gene, binds to hsp90, reducing the affinity and sensitivity of GR to glucocorticoids. Overexpression of FKBP5 is observed in MDD patients, and suicidal MDD patients have decreased orexin levels (84–86).

### 2.6 Oxidative stress

The oxidative and nitrosative stress arising from the incomplete reduction of oxygen at sites of inflammation is associated with depression (87). The superoxide anion, generated in this process, combines with nitric oxide (NO), forming superoxynitrite. This compound can lead to the nitration of cellular constituents, thereby disrupting their functionality and potentially inducing cell demise (88). Augmented production of NO by immune cells is linked to nitrosative stress, marked by an abundance of nitrogen-containing compounds such as nitroxyl ions and NO radicals (89). The research underscores the role of neuronal NO synthase (nNOS) in depression, with evidence indicating that antidepressants can diminish NO levels in individuals with depression (88). Individuals experiencing depression often display decreased levels of non-enzymatic antioxidants and disrupted activity of enzymatic antioxidants such as superoxide dismutase (SOD) and catalase (90). Variations in manganese superoxide dismutase (MnSOD, SOD2) polymorphs are more frequent among those with depression, potentially impacting mitochondrial performance (91). In depression, there is heightened DNA damage marked by increased levels of 8-oxoguanine, likely due to faulty DNA repair systems. These results underscore the importance of oxidative and nitrosative stress in depression development, influencing potential treatments focused on antioxidants (92).

Mitochondrial dysfunction significantly impacts depression by impairing neuronal communication and weakening cellular resilience (93). Mitochondria are critical for cellular energy production, particularly in the brain, which demands a constant ATP supply due to its high energy use and limited storage capacity (94). Research indicates a link between mitochondrial dysfunction and depression, evidenced by decreased mitochondrial respiratory rates and compromised mitochondrial membrane potential. This impairment in mitochondrial function disrupts brain energy metabolism, leading to the emergence of depressive symptoms. Prolonged stress, which is a well-established trigger for depression, causes mitochondrial dysfunction by increasing oxidative stress and inflammation. Depressed patients have higher levels of inflammatory cytokines and oxidative stress markers, connecting these elements to mitochondrial dysfunction and resistance to treatment (95–97). Furthermore, mitochondrial dysfunction leads to the elevated production of reactive oxygen species (ROS) and reactive nitrogen species (RNS), which cause additional damage to cellular structures and intensify depressive symptoms (98). Mitochondria are the main producers of ROS and RNS in cells, and excessive amounts lead to oxidative stress, which harms lipids, proteins, and DNA (99). This oxidative damage is especially critical in the brain because it contains high levels of polyunsaturated lipids, which are susceptible to peroxidation (100). Depressed individuals exhibit increased levels of oxidative stress indicators such as malondialdehyde (MDA), and decreased levels of antioxidants when compared to healthy individuals. This indicates a disparity between pro-oxidants and antioxidants (101). Studies indicate a link between mitochondrial dysfunction and oxidative stress in depression. Using antioxidants has shown potential in alleviating depressive symptoms by reducing oxidative stress and enhancing mitochondrial function (98, 102). For example, supplementing with vitamins A, C, and E has been found to enhance oxidative stress markers and lessen depressive symptoms when used alongside antidepressant treatment (103, 104). Focusing on mitochondria with antioxidant treatments has become a promising therapeutic approach. Antioxidants specifically targeting mitochondria, like MitoQ, have shown to provide protection against oxidative damage (105). Natural antioxidants such as polyphenol compounds have shown potential in mitigating oxidative stress, regulating energy metabolism, and safeguarding brain mitochondria, indicating their promise in depression treatment (106). Thus, impaired mitochondrial function, marked by reduced energy production and elevated oxidative stress, plays a crucial role in the development of depression. Improving mitochondrial health and managing oxidative stress with targeted therapies and antioxidant supplements presents a promising approach for enhancing depression treatment outcomes.

### 2.7 Impaired hippocampal neurogenesis and programmed cell death

Accumulating evidence indicates that impaired hippocampal neurogenesis is a key pathological feature of MDD, particularly within the dentate gyrus, where adult neurogenesis contributes to mood regulation, cognitive flexibility, and stress adaptation (107, 108). Chronic stress and prolonged exposure to glucocorticoids suppress neural stem cell proliferation and neuronal differentiation, leading to reduced hippocampal volume and functional impairments observed in patients with depression (109). Moreover, decreased expression of neurotrophic factors, particularly BDNF, further contributes to impaired neurogenesis and synaptic dysfunction (110, 111).

Apoptosis is one of the most extensively studied forms of programmed cell death in MDD. Increased neuronal apoptosis has been reported in the hippocampus and prefrontal cortex, regions critically involved in emotional regulation (112). Mechanistically, mitochondrial dysfunction leads to cytochrome c release and activation of caspase cascades (caspase-9 and caspase-3), ultimately resulting in neuronal loss and impaired synaptic plasticity (113, 114). Additionally, dysregulation of pro- and anti-apoptotic proteins, such as Bax and Bcl-2, has been observed in depressive conditions (115).

Recent studies have highlighted pyroptosis as a critical inflammatory form of programmed cell death in MDD. Activation of the NLRP3 inflammasome leads to caspase-1 activation and cleavage of gasdermin D, resulting in membrane pore formation and release of pro-inflammatory cytokines such as IL-1β and IL-18 (116–118). Increasing evidence suggests that NLRP3-mediated pyroptosis contributes to neuroinflammation and depressive-like behaviors, establishing a mechanistic link between immune activation and neuronal damage (119, 120).

Autophagy is a critical cellular process responsible for the degradation of damaged organelles and protein aggregates. Dysregulation of autophagy has been increasingly implicated in the pathogenesis of MDD (121). Impaired autophagic flux leads to the accumulation of dysfunctional mitochondria and increased oxidative stress, thereby exacerbating neuronal damage and synaptic dysfunction (122). Key signaling pathways, including mTOR and AMPK, play essential roles in regulating autophagy, and their imbalance has been associated with depressive phenotypes (123).

Ferroptosis, a recently identified form of iron-dependent cell death, is characterized by excessive lipid peroxidation and depletion of glutathione (124). Emerging evidence indicates that ferroptosis contributes to neuronal damage in depression through mechanisms involving iron accumulation, oxidative stress, and mitochondrial dysfunction (125). Reduced activity of glutathione peroxidase 4 (GPX4) and increased lipid ROS have been observed in depressive models, suggesting a potential role of ferroptosis in MDD pathophysiology (126, 127).

Importantly, impaired neurogenesis and programmed cell death pathways are not isolated processes but are highly interconnected. Oxidative stress, neuroinflammation, and mitochondrial dysfunction act as upstream regulators that simultaneously trigger apoptosis, pyroptosis, autophagy imbalance, and ferroptosis, ultimately leading to neuronal loss and impaired neuroplasticity (128, 129). This integrative framework highlights these processes as central mechanistic hubs in MDD and potential targets for therapeutic intervention.

## 3 Role of diet and nutrition in mental health

The field of nutritional psychiatry (NP) has seen significant advancements, with a rising number of studies exploring the effects of diet and nutrients on mental health. NP focuses on using dietary changes and nutrient supplements as clinical tools to prevent and treat psychiatric conditions, supported by growing preclinical and epidemiological evidence (130). With the slowdown in the development of new psychiatric medications and the increasing burden of mental illnesses, NP provides a promising strategy by focusing on changeable factors to enhance mental wellbeing. This strategy should be integrated into a comprehensive model that also encompasses physical activity, sleep management, and other health-enhancing practices (131, 132). Major lifestyle shifts, like unhealthy eating habits and rising stress levels, have resulted in widespread health problems, including mental disorders, costing the global economy trillions of dollars. Consequently, it is crucial to emphasize the role of nutrition in mental health treatment and policy (133). NP investigates the link between diet and mental wellbeing, underscoring the connection between diet and neuropsychiatric conditions (130). Recent initiatives by Nutritional Psychiatry Research (ISNPR) have highlighted that scientifically supported dietary modifications and nutrient-based treatments can significantly enhance mental health. Essential nutrients associated with brain health encompass amino acids, B vitamins, choline, iron, magnesium, omega-3 fatty acids, S-adenosyl methionine, vitamin D, and zinc. NP promotes a return to traditional diets composed of whole foods, emphasizing fruits, lean meats, legumes, nuts, seafood, vegetables, and whole grains, while minimizing processed foods (134). These eating habits promote brain health by offering antioxidant, neurogenesis-promoting, anti-inflammatory, microbiome-altering, and immune-regulating benefits. NP seeks to explore how diet affects mental health and to use specific nutraceuticals to correct nutrient deficiencies and influence neurobiological pathways (135).

The relationship between diet and mental health is a topic of increasing interest, although the scientific data supporting it are still limited and intricate. While popular media frequently asserts a significant connection between what we eat and our mental health, claiming that certain diets can improve mood, cognitive abilities, and even treat neuropsychiatric conditions such as ADHD, autism, and epilepsy, scientific proof for these claims is often insufficient. Consequently, it is difficult to formulate clear dietary guidelines for mental health (136). The brain relies on various nutrients like lipids, amino acids, vitamins, and minerals for its structure and function. Given that diet is a factor we can change, it makes sense that what is eaten can affect brain function and, consequently, mental health (137–139). Nutrients influence the brain in several ways, including direct effects on neuropeptides, neurotransmitters, and the gut microbiota, all of which impact brain function (140–142). Epidemiological research frequently finds links between diet and mental health, but it does not establish a cause-and-effect relationship. Intervention studies, which could provide stronger evidence, often struggle with small sample sizes, diverse study populations, and methodological issues such as challenges in blinding participants and randomizing diets (136). Despite these limitations, there is hope that dietary interventions could have significant effects, particularly in cases of illness or nutrient deficiency. Meta-analyses offer some support for the connection between diet and mental health. For example, following a nutritious diet that includes plenty of fruits, vegetables, fish, and whole grains is linked to a lower likelihood of experiencing depression (143). The Mediterranean diet, specifically, appears to offer protective benefits against depression, though this is not universally supported by all research (144, 145). Randomized controlled trials (RCTs) have indicated that dietary changes can decrease the occurrence of depression, but the results are not uniformly consistent (146). Deficiencies in certain nutrients, such as vitamins B_12_, B_9_, and D, have been connected to cognitive and mental health problems (147, 148). For instance, a lack of vitamin B_12_ is linked to symptoms including fatigue, lethargy, depression, and impaired memory. Although these associations exist, the impact of subclinical deficiencies on mental health is still not well understood (147). Moreover, while elevated serum vitamin D levels have been associated with improved cognitive function and mood, the outcomes of trials involving vitamin D supplementation have been inconsistent (149, 150)The developing field of NP seeks to pinpoint which dietary elements are essential for mental wellbeing and determine the circumstances in which they are most beneficial.

The emerging field of NP is dedicated to identifying the dietary components that are critical for maintaining mental health and understanding the specific conditions under which they are most effective. A key focus of this discipline is the role of polyphenols, which are abundant in fruits, vegetables, tea, and red wine. These compounds have been shown to have anti-inflammatory and antioxidant properties, which can positively influence brain function and mood. Researchers in nutritional psychiatry are investigating how polyphenols and other nutrients can be harnessed to prevent or manage mental health conditions, providing a promising avenue for dietary interventions in mental health care.

## 4 Polyphenols: molecular functions and neuroprotective effects

### 4.1 Definition and classification of polyphenols

**Figure 2:**
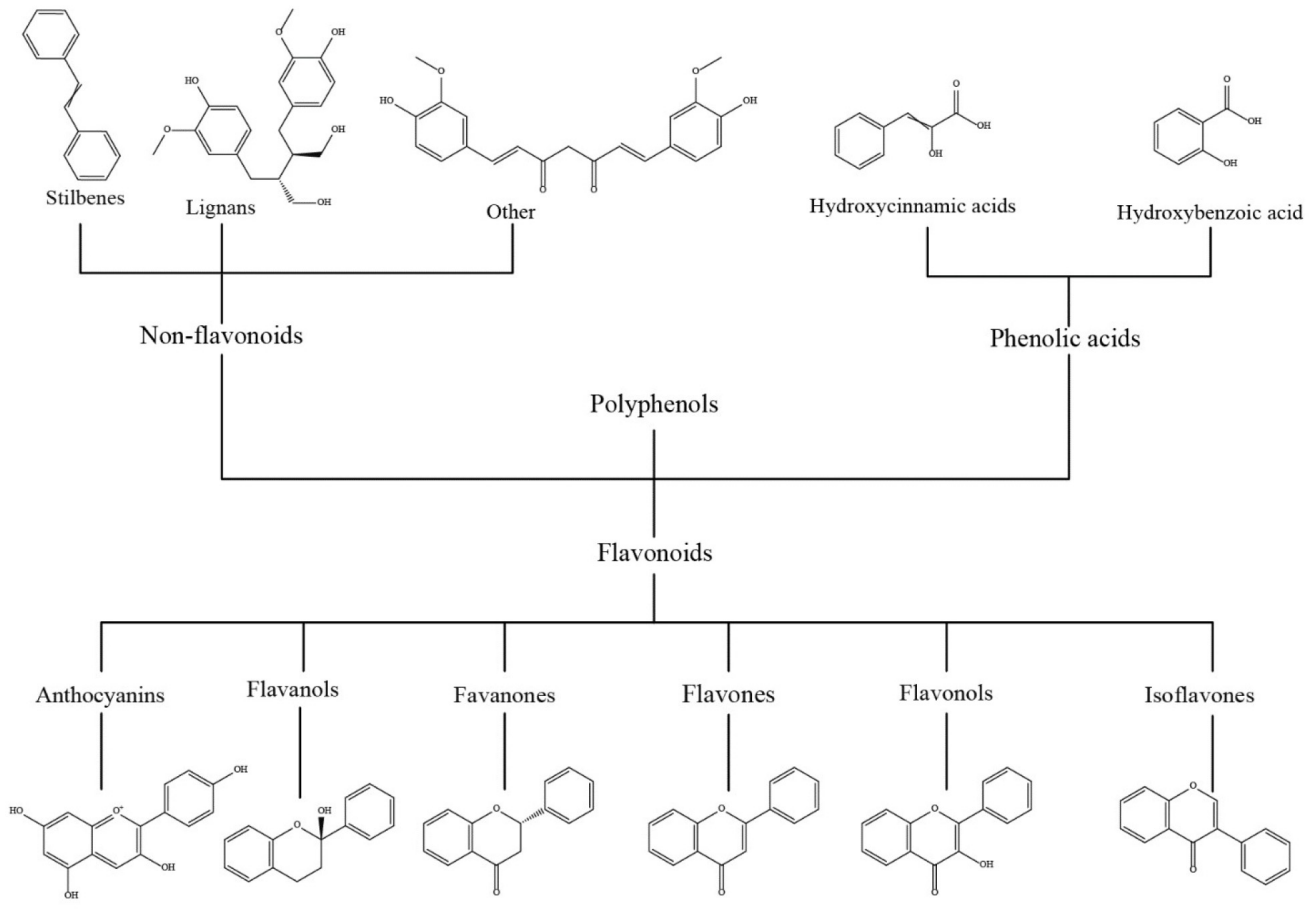
The structure of various polyphenolic compounds.

Polyphenols are naturally occurring compounds produced exclusively by plants as secondary metabolites, with over 8,000 structural variants identified (151). These compounds serve numerous roles, such as shielding plants from stress, filtering ultraviolet radiation, acting as signaling molecules, and contributing to the vibrant colors and aromas of plants (152). Dietary polyphenols are abundant in plant-based foods like fruits, vegetables, and herbs. Chemically, these polyphenols represent a diverse group of compounds, all characterized by a common phenolic structure consisting of hydroxyl groups attached to an aromatic ring (153). Polyphenols are predominantly categorized into flavonoids, phenolic acids, stilbenes, and lignans (Figure 2). These substances, which can be obtained naturally or created synthetically, harness the distinct characteristics of polyphenols for various functional purposes (154). Epidemiological research indicates that consuming more dietary polyphenols is linked to a reduced risk of chronic diseases, including cardiovascular disease, type 2 diabetes, certain cancers, and neurodegenerative conditions (155–157). Polyphenols, characterized by their phenolic hydroxyl groups, possess remarkable antioxidant properties due to their reducing nature (158). In addition to their antioxidant capabilities, research has identified polyphenols for their antibacterial, anti-tumor, immunomodulatory, and anti-radiation effects (159). Additionally, polyphenols are recognized for their outstanding biocompatibility and biodegradability. These characteristics have made them highly valuable in diverse fields such as biomedicine, food health, and cosmetics (160).

### 4.2 Antioxidant activity of polyphenols

The adaptation of organisms to oxygen is closely tied to enzyme function. Enzymes are essential not only for oxygen utilization but also for detoxifying highly reactive metabolites like ROS (161). ROS are highly reactive molecules containing oxygen, such as hydroxyl radicals, superoxide anion radicals, singlet oxygen, and hydrogen peroxide. Oxidative stress occurs when there is a significant imbalance between pro-oxidant and antioxidant activities (162). Excessive ROS production can result in tissue damage and trigger inflammatory processes (163). The antioxidant capabilities of polyphenols are influenced by their functional groups. Hydroxyl groups play a crucial role in antioxidant mechanisms, including free radical scavenging and metal ion chelation (158). Thus, polyphenols possess multiple mechanisms through which they exhibit antioxidant properties. Initially, their inherent antioxidant structure enables them to counteract free radicals directly by donating hydrogen atoms or electrons, effectively reducing their concentration. Moreover, these compounds chelate metal ions, preventing the formation of free radicals catalyzed by redox-active transition metals, thereby enhancing their antioxidant capacity. Additionally, polyphenols can interact synergistically to promote antioxidant activity, facilitating intramolecular hydrogen interactions among them (164–166). Polyphenols have the ability to interact with the non-polar elements within the lipophilic layer of the plasma membrane, impacting the rate of lipid or protein oxidation (167). Certain flavonoids, for example, can impede the penetration of oxidants into the lipophilic core of the plasma membrane, consequently safeguarding its integrity and functionality (168). Polyphenols can also hinder the generation of free radicals by inhibiting enzymes responsible for their production (169). Flavonoids have demonstrated the ability to inhibit the activity of xanthine oxidase, which is recognized as a significant contributor to the production of free radicals (170). Metabolites produced in the xanthine oxidase pathway can worsen oxidative tissue damage, particularly during ischemia when xanthine dehydrogenase converts to xanthine oxidase, resulting in ROS production. Polyphenols have been found to mitigate oxidative damage, thereby reducing xanthine oxidase activity (171). Additionally, flavonoids have been observed to reduce peroxidase activity, consequently restraining the release of free radicals by neutrophils. Moreover, they can promote the activation of these cells through α1-antitrypsin (172). Numerous investigations have revealed the ability of polyphenols to modulate the function of enzymes like cyclooxygenase, lipoxygenase, and nitric oxide synthase (iNOS). By inhibiting these enzymes, polyphenols can effectively reduce the synthesis of arachidonic acid, prostaglandins, NO, and leukotrienes, which are crucial components in the inflammatory process and are responsible for producing metabolites that can exacerbate tissue oxidative damage (173, 174). Polyphenols exhibit the ability to hinder the transcription of inducible iNOS induced by LPS and related pathways in cultured macrophages. This action contributes to the reduction of oxidative damage (175).

### 4.3 Anti-inflammatory activity of polyphenols

Inflammatory cytokines are essential regulators in the body's inflammatory processes, responsible for both triggering and controlling immune responses (176). Persistent and intense inflammation in the body can severely jeopardize an individual's health and wellbeing (177). Recent studies have shown that polyphenols can modulate inflammation (174, 178). Polyphenols can regulate enzymes and signaling systems involved in inflammation, such as tyrosine-protein kinase and threonine-serine protein kinase. These enzymes are known to participate in cellular activation processes, including the activation of B cells, the proliferation of T cells, and the production of cytokines by activated monocytes. Genistein selectively inhibits tyrosine-protein kinase, an enzyme involved in various inflammatory processes (179). Moreover, polyphenolic compounds are recognized for their ability to inhibit transcriptional activity related to inflammation, thereby blocking the expression of pro-inflammatory genes and exhibiting anti-inflammatory effects. It has been revealed that the polyphenol EGCG reduces inflammatory responses by reducing the pro-inflammatory cytokine IL-6 and modulating the anti-inflammatory cytokine TNF-α. EGCG influences inflammation through the NF-kB and PI3K-Akt-mTOR pathways (180). Furthermore, polyphenols like kaempferol notably decrease the levels of pro-inflammatory agents such as COX-2 and inducible iNOS in macrophages stimulated by LPS (181). Additionally, Kaempferol can reduce oxidative stress by decreasing lipid peroxidation and increasing NO production. It also significantly lowers the expression of TNF-α, IL-6, IL-1β, iNOS, and COX-2 (182). Some polyphenols have been shown to influence the secretion of inflammatory cells and prevent neutrophils from releasing β-glucuronidase and lysozyme (183). Additionally, these polyphenols also block the release of arachidonic acid from the plasma membrane. Polyphenols exhibit notable anti-inflammatory properties by scavenging free radicals, regulating the activity of inflammatory cells, and modulating enzymes involved in arachidonic acid metabolism (184).

### 4.4 Neuroprotective effects of polyphenols

Neurodegenerative conditions result from the impairment or depletion of certain neuron clusters, disrupting typical operations within the central nervous system. Typically, these diseases progress unnoticed over extended periods before manifesting clinical indications, emphasizing the significance of preventive measures. While there is a deficiency in effective treatments, several shared mechanisms, such as neuroinflammation and oxidative stress, contribute to numerous neurodegenerative disorders. Researchers are investigating the potential of dietary polyphenols as antioxidants to counteract these processes and provide neuroprotection. Polyphenols found abundantly in dietary sources such as red wine and green tea hold the potential to alleviate neurodegenerative disorders by addressing oxidative stress, inflammation, and various pathways. For instance, epigallocatechin gallate (EGCG) in green tea demonstrates neuroprotective qualities by influencing cellular mechanisms like autophagy and mitochondrial function. Likewise, resveratrol in red wine exhibits neuroprotective effects by diminishing inflammation and improving cognitive abilities. Apart from individual polyphenols, dietary approaches incorporating diverse plant-based substances such as curcumin and *Centella asiatica* offer promise for safeguarding neural health. Additionally, recent investigations propose that polyphenols might influence epigenetic processes, thereby influencing gene expression associated with inflammation and neurodegeneration. Notably, polyphenols have demonstrated the capacity to regulate enzyme activity, such as COX and lipoxygenase, which are crucial in generating inflammatory mediators.

### 4.5 Regulation of neurogenesis and programmed cell death by polyphenols

Recent evidence suggests that the neuroprotective effects of polyphenols extend beyond their antioxidant and anti-inflammatory activities to include the regulation of hippocampal neurogenesis and programmed cell death. These mechanisms are particularly relevant to depression, as impaired neurogenesis and excessive neuronal loss are closely linked to neuroinflammation, oxidative stress, and synaptic dysfunction.

Several polyphenols have shown the ability to support hippocampal neurogenesis under pathological conditions. Curcumin enhanced hippocampal neurogenesis and improved spatial memory after traumatic brain injury through mechanisms involving reduced neuroinflammation and activation of BDNF TrkB PI3K Akt signaling (185). Similarly, caffeic acid alleviated memory deficits and restored neurogenesis related markers in D galactose induced aging rats (186). Epigallocatechin 3 gallate also promoted neuronal survival and increased net hippocampal neurogenesis, partly through PI3K Akt signaling (186). Lycium barbarum polyphenol likewise improved cognitive impairment and enhanced the proliferation and maturation of hippocampal neural cells while reducing oxidative stress through Nrf2 HO 1 signaling (187). However, these effects are not universal, as resveratrol was reported to suppress neural progenitor proliferation and hippocampal neurogenesis in healthy adult mice, indicating that the impact of polyphenols on neurogenesis may depend on biological context and treatment conditions (188).

Polyphenols also modulate several forms of programmed cell death. Their anti apoptotic properties have been linked to reduced oxidative stress, suppression of stress related signaling, and improved cell survival (189, 190). Tea polyphenols, for example, attenuated oxidative stress and apoptosis associated mediators in a toxic injury model (191). In addition, growing evidence indicates that polyphenols can inhibit pyroptosis by targeting inflammasome related pathways. Tea polyphenol nanoparticles blocked gasdermin D dependent pyroptosis in endotoxin induced sepsis, while Qingqiao polyphenols and Echinacea polyphenols reduced inflammasome mediated pyroptotic injury in inflammatory disease models (192–194). Polyphenols have also been recognized as regulators of autophagy, mainly through AMPK, mTOR, SIRT1, and related pathways, although their effects may vary according to disease state and cellular context (195, 196). More recently, anti ferroptotic actions have been described for several polyphenols. Quercetin derived, gallic acid based, catechin based, and tannic acid related systems have all been shown to reduce iron dependent lipid peroxidation and ferroptotic injury in neurological disease models (197–200).

Therefore, these findings indicate that polyphenols act as multifaceted regulators of neuronal plasticity and survival. By influencing neurogenesis and multiple cell death related pathways, including apoptosis, pyroptosis, autophagy, and ferroptosis, they may provide a broader mechanistic basis for their potential antidepressant effects.

## 5 Polyphenols in depression: preclinical evidence

**Figure 3:**
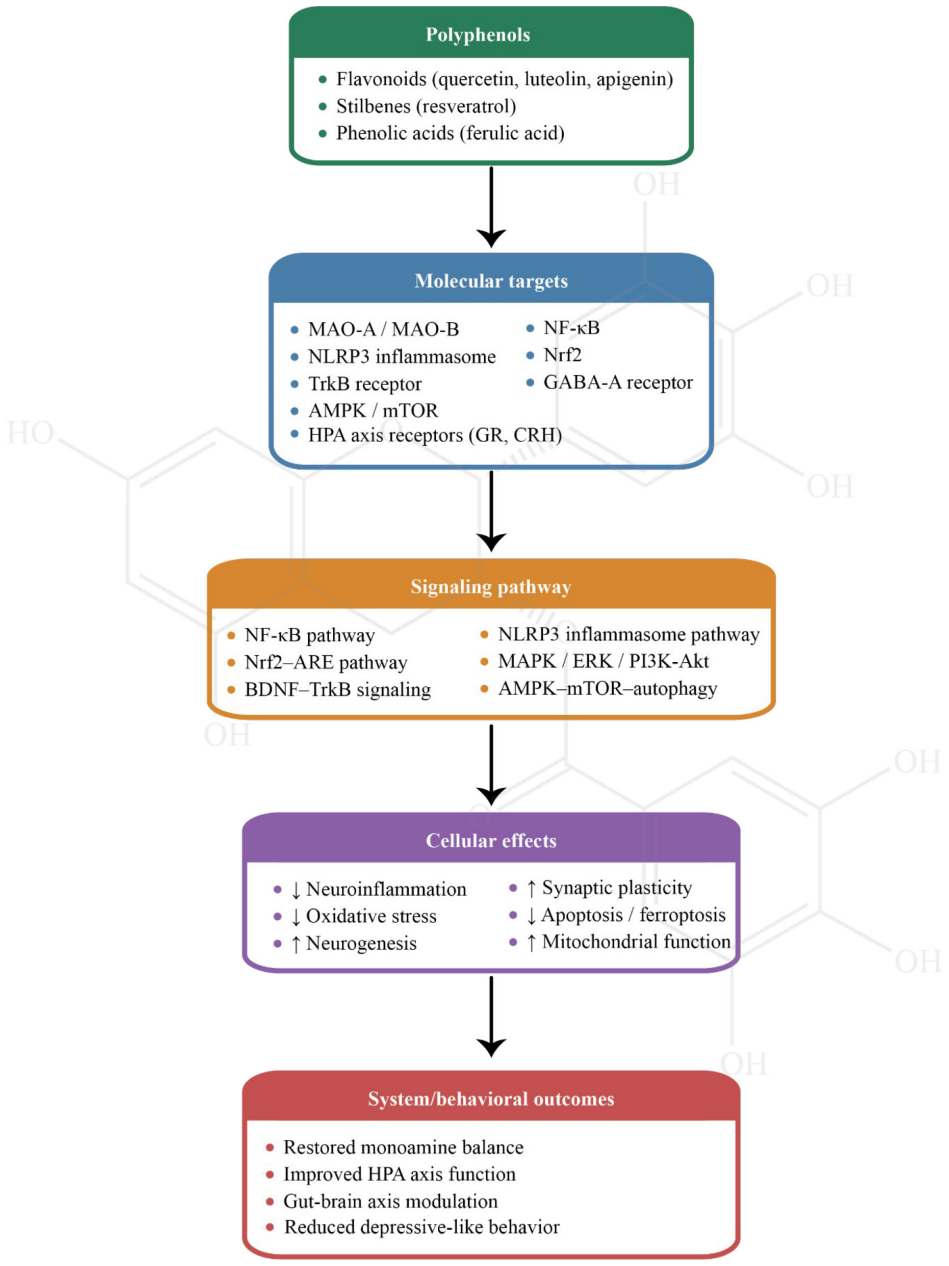
A diagram of anti-depressant mechanisms of polyphenolic compounds.

Various *in vivo* studies have revealed that polyphenol compounds have the potential to exert anti-depressant activity (Table 1). In this section, an overview of various polyphenolic compounds and their antidepressant effects, as demonstrated in preclinical studies, will be provided. The focus will be on the mechanisms through which these compounds exert their therapeutic effects, including anti-inflammatory, antioxidant, neuroprotective, and neurotransmitter-modulating actions (Figure 3). Each subsection will delve into specific polyphenols, detailing their sources, pharmacological properties, and evidence from animal models supporting their potential to alleviate depressive symptoms. This compilation aims to highlight the multifaceted roles of polyphenols in depression treatment, offering insights into their promise as therapeutic agents.

**Table 1:** An overview of anti-depressant effects of various polyphenols considered in the pre-clinical studies.

Type of polyphenol	Animal species	Depression induction method	Dose and duration	Administration route	Behavioral test	Main behavioral and molecular effects (directional)	Ref.
**Curcumin**	Male Sprague-Dawley rats	CUMS	0–100 mg/kg/day, 28 days	Oral gavage	SPT, FST, NSFT, OFT	Reduced depression-like behavior; decreased oxidative stress markers and upregulated Nrf2–ARE signaling.	Liao et al. (204)
	Adult female albino rats	Ovariectomy	0–100 mg/kg/day, 30 days	Oral administration	OFT	Improved open-field performance; downregulated monoamine oxidase-B and upregulated tyrosine hydroxylase in limbic regions; modulated dopamine and norepinephrine levels.	Saied et al. (309)
	Male Swiss mice	CUMS	0–50 mg/kg/day, 28 days	Oral gavage	FST, OFT, Elevated plus maze	Reduced immobility and anxiety-like behavior; decreased oxidative stress and enhanced antioxidant activity.	da Silva Marques et al. (205)
**Curcumin NPs**	Female C57BL/6 mice	CORT	0–20 mg/kg/day, 14 days	Intraperitoneal injection	FST, rotarod test	Reduced CORT-induced apoptosis; increased dopamine release; upregulated CB1, p-MEK1, and p-ERK1/2 expression.	He et al. (310)
	C57BL/6 mice	CORT	0–20 mg/kg/day, 14 days	Oral gavage	FST, rotarod test	Improved depressive-like behavior; increased dopamine and serotonin release; reduced apoptotic cell death; upregulated CB1 expression.	He et al. (311)
	Female CBR1+/+ and CBR1–/– mice	CORT	0–20 mg/kg/day, 2 days	Intraperitoneal injection	FST	Increased mature neuronal markers, dopamine and norepinephrine release, and CBR1-related gene expression; improved depressive-like behavior.	He et al. (312)
	Male Sprague-Dawley rats	LPS	0–40 mg/kg/day, 7 days	Intraperitoneal injection	FST, TST	Reduced depression-like behavior; downregulated brain p-NF-κB, TNF-α, and COX-2 expression.	Rubab et al. (208)
**Curcumin derivates**	Male ICR mice	Not specified	0–10 mg/kg/day, 0–1 day	Oral gavage	FST, TST, LAT	Reduced immobility; activated the 5-HT1A/cAMP/PKA/CREB/ BDNF pathway.	Lian et al. (313)
	ICR male mice	Not specified	0–10 mg/kg/day, 3 days	Oral administration	FST, TST, LAT, OFT, NSFT, SPT	Reduced depression- and anxiety-like behaviors; suppressed MAO-A activity and increased brain monoamine levels.	Pan et al. (206)
Quercetin	Male Wistar rats	Olfactory bulbectomy	0–80 mg/kg/day, 14 days	Oral gavage	OFT, FST	Improved behavioral deficits; reduced microglial activation, oxidative-nitrosative stress, and neuroinflammation/apoptotic signaling.	Rinwa and Kumar (219)
	Wistar rats	Adriamycin	0–60 mg/kg/day, 0–1 day	Intraperitoneal injection	FST, OFT, EPM	Ameliorated Adriamycin-induced neurobehavioral and immune alterations.	Merzoug et al. (314)
	Female Swiss mice	Bilateral olfactory bulb destruction	0–30 mg/kg/day, 14 days	Oral administration	FST, TST, OFT, splash test	Reduced depression-like behavior; inhibited NMDA receptor and/or nitric oxide-related signaling; lowered hippocampal lipid hydroperoxide levels.	Holzmann et al. (315)
	Swiss albino mice	CUMS	0–30 mg/kg/day, 21 days	Oral administration	EPM, OFT, SPT	Improved depressive-like behavior; reduced oxidative stress and pro-inflammatory cytokines; protected hippocampal neurons.	Mehta et al. (220)
	Male ICR mice	Predatory stress	0–50 mg/kg/day, 28 days	Intraperitoneal injection	TST	Reduced anxiety-, fear-, and depression-like behaviors after repeated predator stress.	Anggreini et al. (316)
	Male Swiss albino mice	CUMS	0–25 mg/kg/day, 28 days	Oral gavage	FST, TST, OFT	Reduced depression-like behavior; decreased inflammatory and oxidative stress-related changes; increased 5-HT levels.	Khan et al. (317)
	Sprague-Dawley rats	Maternal separation stress	0–30 mg/kg/day, 56 days	Quercetin-enriched diet	EPM, OFT, FST	Reduced depressive-like behavior; restored HPA-axis function and BDNF levels; modulated the microbiota–gut–brain axis.	Donoso et al. (221)
	Male Sprague-Dawley rats	LPS	0–40 mg/kg/day, 14 days	Intragastric administration	SPT, OFT, FST, MWM	Improved depressive-like behavior and cognition; regulated hippocampal and prefrontal cortex BDNF-related Copine 6 and TREM1/2 expression.	Fang et al. (318)
	Adult male Wistar albino rats	CUMS	0–30 mg/kg/day, 35 days	Intraperitoneal injection	FST, SPT, LAT	Reduced depression-like behavior; attenuated oxidative stress and inflammatory changes associated with chronic stress.	Sahin et al. (319)
	C57BL/6N mice	Chronic social defeat stress	0–2 g/kg/diet, 14 days	Quercetin-enriched diet	OFT, EPM, TST, SPT, social interaction	Reduced stress-related behavioral abnormalities; suppressed A1 astrocyte reactivity in the mPFC and hippocampus.	Zhang et al. (320)
	SPF-grade male Sprague-Dawley rats	CUMS	0–30 mg/kg/day, 56 days	Oral gavage	SPT	Improved depressive-like behavior; reduced oxidative stress and inflammation; modulated serum neurochemical-related elements.	Guan et al. (321)
	Male ICR mice	CUMS	0–30 mg/kg/day, 28 days	Intragastric administration	SPT, OFT, TST	Reduced depressive-like behavior; promoted adult hippocampal neurogenesis via the FoxG1/CREB/BDNF pathway.	Ma et al. (222)
	Female C57BL/6J mice	Estrogen receptor α deficiency	0–100 mg/kg/day, 70 days	Oral administration	OFT, FST, TST	Reversed depression-like behavior; activated hippocampal and cardiac BDNF–TrkB–AKT/ERK1/2 signaling.	Wang et al. (223)
	Male C57BL/6J mice	CORT	0–80 mg/kg/day, 14 days	Oral gavage	OFT, FST, EPM	Reduced depressive-like behavior; downregulated caspase-3 and upregulated VEGF and BDNF; reduced neuroinflammation and oxidative damage.	Ge et al. (224)
	Swiss albino mice	CUMS	0–20 mg/kg/day, 0–21 days	Oral administration	FST, OFT	Reduced depression-like behavior; lowered brain oxidative stress; restored serotonin levels, likely via MAO-A inhibition.	Singh et al. (322)
Resveratrol	Male ICR mice	Despair tasks	0–80 mg/kg/day, 3 days	Oral gavage	FST, TST, LAT	Reduced immobility; increased serotonin and noradrenaline levels; inhibited MAO-A activity.	Xu et al. (323)
	Male Sprague-Dawley rats	CUMS	0–80 mg/kg/day, 21 days	Intragastric administration	SPT, Shuttle-box	Improved depressive-like behavior; increased serotonin and noradrenaline; suppressed MAO-A activity in relevant brain regions.	Yu et al. (324)
	Male Wistar rats	Reserpine	0–60 mg/kg/day, 3 days	Oral administration	OFT, SFT	Produced antidepressant-like effects comparable to fluoxetine.	Ahmed et al. (325)
	Male WKY rats	Genetic depression-prone strain	0–40 mg/kg/day, 7 days	Intraperitoneal injection	LAT, FST, SPT	Increased hippocampal BDNF; increased sucrose consumption; reduced FST immobility.	Hurley et al. (326)
	Male Wistar rats	CUMS	0–80 mg/kg/day, 35 days	Intraperitoneal injection	SPT, FST, OFT	Reduced depressive-like behavior; normalized corticosterone; upregulated hippocampal and amygdalar pERK, pCREB, and BDNF.	Liu et al. (327)
	Male C57BL/6J mice	Chronic constriction injury	0–60 mg/kg/day, 21 days	Oral administration	LAT, SFT	Reduced neuropathic depression-like behavior; enhanced serotonergic activity.	Zhao et al. (328)
	Male Swiss albino mice	CORT	0–80 mg/kg/day, 21 days	Subcutaneous injection	TST, SPT, FST	Reduced depressive-like behavior; normalized HPA-axis activity; increased hippocampal BDNF.	Ali et al. (329)
	Male Kun-Ming mice	LPS	0–80 mg/kg/day, 7 days	Intraperitoneal injection	TST, SPT, LAT, FST	Reduced LPS-induced depressive-like behavior; downregulated inflammatory signaling and restored pCREB/BDNF expression in the PFC and hippocampus.	Ge et al. (330)
	Male Wistar rats	CUMS	0–80 mg/kg/day, 28 days	Intraperitoneal injection	SPT, FST, OFT	Reversed depressive-like behavior; upregulated phospho-Akt and mTOR in hippocampus and PFC.	Liu et al. (331)
	Male Wistar rats	CRS	0–80 mg/kg/day, 21 days	Intraperitoneal injection	OFT, FST, SPT	Reversed stress-induced depression; normalized hippocampal and prefrontal BDNF, pERK, Bcl-2, and Bax levels.	Wang et al. (230)
	Male ICR mice	LPS	0–0.3 mg/kg/day, 1 day	Intraperitoneal injection	SPT, FST	Reduced depression-like behavior; prevented hippocampal mitochondrial dysfunction and reduced oxidative stress and apoptosis.	Chen et al. (231)
	Male adult Sprague-Dawley rats	CUMS	0–15 mg/kg/day, 21 days	Intragastric administration	SPT, OFT, FST	Reduced depressive-like behavior; regulated HPA-axis function; modulated BDNF, IL-6, CRP, TNF-α, and Wnt/β-catenin signaling.	Yang et al. (332)
	Male Wistar rats	CUMS	0–80 mg/kg/day, 56 days	Oral administration	SPT	Reduced depressive-like behavior; decreased neuroinflammation, oxidative stress, and apoptosis; upregulated hippocampal BDNF and β-catenin.	Abd El-Fattah et al. (333)
	Male SPF-grade C57BL/6 mice	Social isolation + CUMS	0–30 mg/kg/day, 21 days	Intraperitoneal injection	FST, TST, SPT	Reduced depressive-like behavior; increased prefrontal dopamine and serotonin; enhanced neuropeptide expression.	Gu et al. (334)
	Female C57BL/6J mice	Ovariectomy	0–20 mg/kg/day, 14 days	Intraperitoneal injection	OFT, EPM, MWM, TST, FST	Improved psychobehavioral deficits; upregulated hippocampal Sirt1 and downregulated inflammatory cytokines.	Liu et al. (232)
	Male Sprague-Dawley rats	CUMS	0–80 mg/kg/day, 28 days	Intraperitoneal injection	SPT, FST, OFT	Reversed depressive-like behavior; activated Akt/GSK3β signaling; reduced pro-inflammatory cytokines and apoptosis-related changes.	Shen et al. (335)
	Male ICR mice	CUMS	0–200 mg/kg/day, 28 days	Oral gavage	SPT, FST	Reduced depressive-like behavior; increased hippocampal ATP and mitochondrial biogenesis; regulated serotonin and GAP-43 expression.	Shen et al. (233)
	Adult male Wistar albino rats	CUMS	0–30 mg/kg/day, 35 days	Intraperitoneal injection	FST, SPT, LAT	Reduced depressive-like behavior; protected against stress-related oxidative and inflammatory changes.	Sahin et al. (319)
Ferulic acid	Male Swiss mice	TST stress model	0–10 mg/kg/day, 60 min	Oral administration	TST, OFT, FST	Reduced immobility; modulated the serotonergic system.	Zeni et al. (336)
	Male ICR mice	Reserpine injections	0–80 mg/kg/day, 30 min	Intraperitoneal injection	Thermal hyperalgesia, mechanical allodynia, TST, FST, LAT	Reversed depression- and pain-like behaviors; restored monoamines; reduced oxidative stress, inflammatory mediators, and apoptosis-related signaling.	Xu et al. (252)
	Male ICR mice	Unavoidable/ inescapable stress	0–80 mg/kg/day, 30 min	Oral administration	FST, TST, LAT	Reduced immobility; increased hippocampal and cortical serotonin and norepinephrine; likely inhibited MAO-A.	Chen et al. (337)
	Male Swiss mice	TST and FST stress	0–10 mg/kg/day, 21 days	Oral administration	TST, OFT, FST	Reduced depression-like behavior; increased antioxidant enzyme activity and reduced lipid peroxidation.	Lenzi et al. (338)
	Male ICR mice	TST and FST stress	0–90 mg/kg/day, 30 days	Oral administration	TST, LAT, FST	Enhanced monoaminergic function; increased serotonin and noradrenaline, particularly with piperine co-administration.	Li et al. (339)
	Male ICR mice	CUMS	0–80 mg/kg/day, 28 days	Oral administration	SPT, FST	Reduced depressive-like behavior; upregulated BDNF, PSD95, and synapsin I in the brain.	Liu et al. (250)
	Male Swiss albino mice	Epilepsy induction	0–80 mg/kg/day, 15 days	Oral administration	TSAT, SPT	Improved comorbid depression-like behavior; restored corticosterone; reduced IL-1β, TNF-α, and brain indoleamine 2,3-dioxygenase activity.	Singh et al. (254)
	Male Swiss mice	CORT	0–1 mg/kg/day, 7 days	Oral administration	TST, OFT, splash test	Reversed behavioral and oxidative stress changes induced by CORT; suggested normalization of HPA-axis-related dysfunction.	Zeni et al. (251)
	Male ICR mice	TST stress model	0–5 mg/kg/day, 7 days	Oral administration	TST	Upregulated limbic genes related to cell survival, energy metabolism, and dopamine synthesis.	Sasaki et al. (340)
	Male Sprague-Dawley rats	Prenatal stress	0–50 mg/kg/day, 28 days	Intragastric administration	SPT, FST, OFT	Reduced depressive-like behavior in offspring; modulated inflammatory pathways and HPA-axis function.	Zheng et al. (261)
Catechins	Male Wistar rats	CUMS	0–50 mg/kg/day, 28 days	Oral administration	SPT, OFT	EGCG reduced depressive-like behavior; decreased colonic serotonin and increased hippocampal serotonin.	Li et al. (244)
	Male Wistar rats	CUMS	0–20 mg/kg/day, 7 days	Intragastric administration	SPT, OFT	EGCG reduced depressive-like behavior; decreased hippocampal IL-6 and NO; downregulated caspase-3 and caspase-9.	Wang et al. (242)
	Female Sprague-Dawley rats	Ovariectomy	0–400 mg/kg/day, 28 days	Oral administration	MWM	EGCG improved postmenopausal depressive/cognitive changes; increased hippocampal BDNF and synaptic plasticity.	Ko et al. (246)
	Male C57BL/6 mice	CMS	0–2 mg/kg/day, 35 days	Oral gavage	SPT, OFT	Epicatechin reduced anhedonia and anxiety-like behavior; modulated kynurenine aminotransferases to promote stress resilience.	Martinez-Damas et al. (240)
	Male Swiss albino mice	CUMS	0–200 mg/kg/day, 35 days	Oral gavage	FST	EGCG reduced depressive-like behavior; downregulated IL-1β; upregulated hippocampal BDNF mRNA; reduced CA3 neuronal lesions.	Abdelmeguid et al. (239)
	SPF male C57BL/6J mice	CUMS	0–25 mg/kg/day, 42 days	Intragastric administration	SPT, FST	EGCG reduced depressive-like behavior; suppressed NLRP3 inflammasome activation; inhibited mTOR signaling; restored autophagy; reduced apoptosis markers.	Zhang et al. (241)
	Swiss male albino mice	CUMS	0–25 mg/kg/day, 21 days	Intraperitoneal injection	TST, LAT	EGCG nanoparticles improved antidepressant-like behavior; reduced brain MAO-A, malondialdehyde, and catalase; increased brain glutathione.	Dahiya et al. (341)
p-Coumaric acid	Male Sprague-Dawley rats	LPS	0–200 mg/kg/day, 1 day	Intraperitoneal injection	TST, FST, SPT	Reduced depressive-like behavior; reduced inflammatory cytokines; prevented BDNF downregulation; blocked LPS-induced LTD.	Lee et al. (342)
	Male ICR mice	CORT	0–75 mg/kg/day, 1 day	Subcutaneous injection	SPT, FST, LAT, MWM	Reduced depression-like behavior and memory impairment; downregulated AGE-RAGE-mediated inflammatory signaling; reduced IL-1β and TNF-α.	Yu et al. (276)
	Male C57BL/6J mice	CRS	0–100 mg/kg/day, 21 days	Oral administration	FST	Reduced depressive-like behavior; modulated neurotransmitters and stress hormones; activated relevant intracellular signaling pathways.	Oh et al. (275)
	Male ICR mice	LPS	0–75 mg/kg/day, 2 days	Intraperitoneal injection	NOR, TST, MWM	Improved depressive-like behavior and cognition; restored PKA-CREB-BDNF signaling; reduced hippocampal pro-inflammatory cytokines.	Cao et al. (343)
Genistein	Adult male albino mice	LAT, TST, FST paradigms	0–10 mg/kg/day, 10 days	Oral administration	LAT, TST, FST	Produced synergistic antidepressant-like effects with amitriptyline.	Gupta et al. (344)
	Male ICR mice	FST and TST paradigms	0–45 mg/kg/day, 21 days	Oral administration	FST, TST, LAT	Reduced depression-like behavior; increased brain monoamines; inhibited MAO activity via a 5-HT1A-dependent mechanism.	Hu et al. (279)
	BALB/c mice	CUMS	0–5 mg/kg/day, 49 days	Intraperitoneal administration	Nest building, splash test	Reduced depressive-like behavior; downregulated miR-221/222 and modulated Cx43 in the prefrontal cortex.	Shen et al. (345)
	Male albino Wistar rats	CMS	0–100 mg/kg/day, 45 days	Oral administration	FST, OFT	Reduced depressive-like behavior; increased BDNF mRNA/protein and monoamines; decreased serum cortisol.	Chang et al. (346)
Tannic acid	BALB/c female mice	CMS	0–100 mg/kg/day, 49 days	Intraperitoneal administration	FST, TST, SPT	Reduced depressive-like behavior; regulated neurotransmitters, cortisol, acetylcholinesterase, BDNF, and oxidative stress parameters.	Chandrasekhar et al. (347)
	Male Swiss mice	LPS	0–60 mg/kg/day, 7 days	Oral administration	Splash test, FST, TST, OFT	Reduced LPS-induced depressive-like behavior; lowered oxidative stress, lipid peroxidation, and brain TNF-α.	Luduvico et al. (348)
Apigenin	Male ICR mice	CMS	0–14 mg/kg/day, 28 days	Oral administration	FST	Reduced immobility; reversed sucrose preference deficits; modulated neurotransmitter levels.	Yi et al. (349)
	Male ICR mice	LPS	0–50 mg/kg/day, 7 days	Intraperitoneal administration	TST, SPT, OFT	Reduced LPS-induced depressive-like behavior; downregulated pro-inflammatory cytokines and NF-κB signaling.	Li et al. (267)
	Male ICR mice	CORT	0–40 mg/kg/day, 21 days	Subcutaneous injection	SPT, FST	Reduced depressive-like behavior; upregulated hippocampal BDNF.	Weng et al. (271)
	Male BALB/c mice	CRS	0–60 mg/kg/day, 14 days	Oral gavage	SPT, OFT, FST	Reduced depressive-like behavior; enhanced autophagy via the AMPK/mTOR pathway.	Zhang et al. (350)
	Albino mice	TST and FST paradigms	0–50 mg/kg/day, 1 day	Oral administration	TST, FST	Reduced immobility through α-adrenergic, dopaminergic, and 5-HT3 receptor-related mechanisms.	Al-Yamani et al. (351)
	Male mice	Streptozotocin	0–40 mg/kg/day, 1 day	Intraperitoneal injection	Splash test, OFT, FST	Reduced depressive-like behavior; regulated AMPK-related energy homeostasis; downregulated Nlrp3 and Tlr4 inflammatory signaling.	Bijani et al (269)
	Albino mice	CMS	0–50 mg/kg/day, 21 days	Oral administration	TST, LAT, SPT	Reduced immobility; increased sucrose preference; reduced corticosterone and oxidative stress markers.	Alghamdi et al. (270)
	Male Swiss mice	CUMS	0–25 mg/kg/day, 14 days	Intraperitoneal administration	Splash test, EPM, TST, FST, SST	Reduced depression-like behavior; inhibited MAO-A activity; reduced oxidative stress and neuroinflammatory signaling.	Olayinka et al. (352)

SPT, Sucrose preference test; FST, Forced swimming test; NSFT, Novelty-suppressed feeding test; OFT, Open field test; TST, Tail suspension test; LAT, Locomotor activity test; EPM, Elevated plus maze; MWM, Y-maze and Morris water maze; NOR, Novel object recognition; CMS, Chronic mild stress; CORT, corticosterone; LPS, Lipopolysaccharide; CRS, Chronic restraint stress.

### 5.1 Curcumin

Curcumin is a polyphenolic compound derived from turmeric (*Curcuma longa*), recognized for its diverse pharmacological effects including anti-inflammatory, antimicrobial, antioxidant, antiangiogenic, and anti-proliferative properties (201). Moreover, the antidepressant effects of curcumin have been documented in models of major depression (202). The antidepressant potential of curcumin is linked to various mechanisms, such as its anti-inflammatory actions, neuroprotective properties, enhancement of neurotransmitters (serotonin and dopamine), and inhibition of MAO (203). In a study, curcumin exhibited antidepressant properties by reversing behavioral alterations in rats subjected to chronic unpredictable mild stress (CUMS). It enhanced sucrose preference, shortened immobility duration in the forced swim test (FST), and alleviated anxiety-like behaviors. Additionally, curcumin reduced CORT levels, decreased oxidative stress markers such as MDA and Nox2, and increased antioxidant enzyme activity (e.g., catalase). Moreover, curcumin stimulated the Nrf2-ARE pathway, leading to elevated levels of nuclear Nrf2 and its downstream targets (NQO-1 and HO-1). It also boosted synaptic plasticity by increasing the expression of BDNF, PSD-95, and synaptophysin. These findings indicated that curcumin's antidepressant effects are likely due to its regulation of oxidative stress, enhancement of neuroplasticity, and activation of Nrf2 signaling (204). In a separate study, curcumin demonstrated strong antidepressant effects by reducing anxiety and hyperactivity in animal models subjected to stress. It bolstered antioxidant defenses, specifically enhancing catalase activity in the brain, which suggests protection against oxidative stress. The observed reduction in floating times in the FST indicated the effectiveness of curcumin in alleviating depressive symptoms. Furthermore, curcumin demonstrated potential as an adaptogen, enhancing both physical and cognitive resilience during periods of stress. Therefore, curcumin presents itself as a safe and efficient option for treating depression and associated conditions, underscoring its promise as a therapeutic nutraceutical (205). J147, derived from curcumin, demonstrated notable antidepressant effects in mice by decreasing immobility in assessments such as the FST and Tail Suspension Test (TST) after brief administration. Additionally, it displayed anxiolytic properties in the Open Field Test (OFT), enhancing exploratory behavior comparable to diazepam. In terms of its mechanisms, J147 elevated serotonin and noradrenaline levels in the frontal cortex and hippocampus, alongside suppressing the activity of MAO-A. These effects indicate that J147 boosts neurotransmitter levels, which likely underlies its therapeutic benefits against depression and anxiety (206). Curcumin nanoparticles (Cur-IONPs) exhibited antidepressant effects in a rat depression model by restoring swimming behavior to near-normal levels and decreasing immobility. They balanced oxidative stress markers in the brain and regulated enzyme activities linked to depression. Cur-IONPs also enhanced levels of important neurotransmitters in the brain and displayed antioxidant properties, indicating their potential as a beneficial therapy for depression due to their diverse therapeutic effects (207). Curcumin NPs (Cur-NLCs) showed antidepressant effects in rats with LPS-induced depression. This was indicated by their capacity to enhance active behaviors such as struggling and decrease passive behaviors like immobility in tests involving the forced swim and tail suspension tests. Additionally, Cur-NLCs displayed anxiolytic effects in models like the light-dark box and elevated plus maze, where they promoted exploration in areas typically avoided by rodents. Histological examination demonstrated that Cur-NLCs attenuated neurodegeneration induced by LPS in the cortex and hippocampus, maintaining the structural integrity of neurons and overall tissue architecture. Moreover, Cur-NLCs alleviated neuroinflammation by decreasing the expression of inflammatory markers such as p-NF-κB, TNF-α, and COX-2 in the brain. These findings underscore the potential of Cur-NLCs as promising neuroprotective agents for the treatment of depression and associated neurological conditions (208).

Together, these findings show that curcumin influences several core pathways that are central to the development and persistence of depressive symptoms. Curcumin consistently reduces inflammatory signaling by lowering the expression of cytokines such as IL-1β, IL-6, and TNF-α, and by suppressing the activation of NF-κB and related mediators. This direct anti-inflammatory action aligns with the prominent role of neuroimmune disturbances in depression (209, 210). Curcumin also counteracts oxidative stress by reducing lipid peroxidation, restoring antioxidant enzyme activity, and activating pathways such as Nrf2, which enhances cellular defense against oxidative injury. These antioxidant effects support neuronal health and reduce stress-related cellular damage (204, 211). In addition, curcumin enhances neuroplasticity by increasing the expression of BDNF, synaptic proteins, and signaling molecules that promote neuronal growth and resilience. By improving neuroplastic signaling, curcumin addresses one of the major neurobiological deficits observed in depressive disorders (212, 213). Curcumin also modulates neurotransmitter systems by increasing serotonin, dopamine, and noradrenaline levels, and by inhibiting monoamine oxidase. This improvement in neurotransmission contributes to its antidepressant-like behavioral effects (214).

Curcumin further influences stress physiology by lowering circulating corticosterone, normalizing hypothalamic and pituitary stress hormones, and improving the functional integrity of neural circuits disrupted by prolonged stress exposure. These actions are consistent with the established involvement of endocrine dysregulation in depressive symptoms (215, 216). There is also growing evidence that curcumin interacts with the gut–brain axis by reducing systemic inflammation, improving microbial balance, and strengthening barrier function, suggesting additional pathways through which curcumin may exert mood-regulating effects (217). Therefore, these diverse but interconnected mechanisms indicate that curcumin targets several biological domains that are relevant to depressive pathology. Its combined effects on inflammation, oxidative stress, neuroplasticity, neurotransmission, endocrine regulation, and gut–brain communication provide a coherent rationale for its emerging role as a promising therapeutic candidate for depression.

### 5.2 Quercetin

Quercetin, a widely recognized flavonoid, possesses diverse biological activities including antioxidant, anti-inflammatory, anti-apoptotic, neuroprotective, and cardioprotective properties (218). The earlier study demonstrated that quercetin exhibited antidepressant effects by reducing the neuroinflammatory response of microglia in rats undergoing olfactory bulb resection (219). Quercetin demonstrated antidepressant properties and mechanisms in mice exposed to chronic unpredictable stress (CUS). Through various behavioral assessments including the Elevated Plus Maze (EPM), Open Field Test (OFT), Sucrose Preference Test (SPT), and Passive Avoidance Step-Through Task (PAST), quercetin reversed behaviors indicative of anxiety, restored preferences for sucrose, enhanced locomotor activity, and improved memory performance. Quercetin mechanistically alleviated stress-induced alterations in behavior by influencing the FoxG1/CREB/BDNF signaling pathway, which supports neurogenesis and neurotransmitter functions associated with mood regulation. Moreover, quercetin reduced oxidative stress in the hippocampus through the restoration of antioxidant levels and mitigation of oxidative/nitrosative stress markers, thereby safeguarding hippocampal neurons against damage induced by stress. In addition, quercetin inhibited hippocampal inflammation by reducing the expression of pro-inflammatory cytokines (TNF-α, IL-6, IL-1β, COX-2), thereby enhancing its neuroprotective effects against neurological complications induced by stress. These results suggest that quercetin holds promise as a therapeutic candidate for mitigating depression and related cognitive impairments triggered by chronic stress (220). Quercetin and various polyphenols were investigated for their potential to alleviate depression in rats that experienced early life stress. Quercetin notably decreased symptoms of depression as assessed by tests such as the forced swim test, while also demonstrating anxiolytic properties in the elevated plus maze. Polyphenols also impacted the diversity of gut microbiota, modifying the composition of bacteria and preventing decreases in propionate levels. These results indicate that polyphenols might mitigate depression induced by stress through their influence on neurochemical pathways and gut microbiota (221). Quercetin successfully mitigated depressive-like behaviors triggered by prolonged stress in mice. It countered weight loss, enhanced preference for sucrose, decreased immobility in behavioral assessments, and enhanced locomotor activity. Additionally, quercetin stimulated the generation of new neurons in the adult hippocampus, contributing to its therapeutic effects. Quercetin functioned through the FoxG1/CREB/BDNF signaling pathway to promote neuronal growth and neurotransmitter activities linked to mood regulation. These results propose that quercetin holds potential as a viable therapy for depression by bolstering brain health and functionality (222). Quercetin demonstrated the ability to alleviate symptoms resembling depression and improve heart function in female mice lacking ERα. The research indicated that quercetin reduced the duration of immobility in behavioral tests such as the TST and FST. Additionally, quercetin reduced systolic blood pressure and activated key proteins involved in neuronal and cardiac function, including BDNF, TrkB, AKT, and ERK1/2, within the hippocampus and heart tissues. These findings suggest that quercetin exerts significant antidepressant and cardioprotective effects by influencing the BDNF-TrkB-AKT/ERK1/2 pathways, thereby ameliorating hippocampal and cardiac dysfunctions induced by ERα deficiency in female mice (223).Quercetin, a natural flavonoid, demonstrates promising antidepressant effects in animal models exposed to chronic stress. It reduces depressive-like behaviors in tests like the FST and EPM, suggesting mood improvement under stress conditions. Mechanistically, quercetin acts through anti-inflammatory pathways by decreasing pro-inflammatory cytokines in the brain (IL-1β, IL-6, and TNF-α), which are linked to depression. It also exerts antioxidant effects, protecting neurons from oxidative damage and supporting brain health. Additionally, quercetin enhances neurotrophic factors like BDNF and VEGF, which are crucial for neuronal function and mood regulation. These actions collectively highlight quercetin's potential as a therapeutic agent for depression, meriting further clinical investigation (224).

Beyond its antioxidant and anti-inflammatory effects, quercetin also exerts meaningful actions on the gut–brain axis, a pathway increasingly implicated in depressive disorders. Preclinical studies show that quercetin helps restore microbial diversity that becomes disrupted under chronic stress, particularly by preventing reductions in SCFA producing bacteria (225). These bacteria contribute to gut barrier integrity and help suppress systemic inflammation. By preserving these microbial populations, quercetin reduces gut permeability, limits the leakage of bacterial components such as lipopolysaccharide into the bloodstream, and decreases peripheral immune activation (226). This sequence of events ultimately reduces circulating cytokines that influence brain function and mood. Quercetin's ability to stabilize the gut barrier and support SCFA availability thus connects directly to one of the central biological pathways that contribute to the development of depressive symptoms.

Quercetin also interacts with neurobiological mechanisms in the brain. Its activation of the CREB and BDNF signaling cascade helps counteract the loss of neuroplasticity often observed in chronic stress and depressive states. By enhancing BDNF expression and promoting hippocampal neurogenesis, quercetin supports the structural and functional adaptations required for mood regulation (222). In parallel, quercetin suppresses key inflammatory mediators such as IL-1β, IL-6, TNF-α, COX-2, and inducible nitric oxide synthase, thereby reducing neuroinflammation that contributes to neuronal dysfunction (227, 228). Through these combined actions on gut microbiota, neuroinflammatory pathways, and neuroplasticity, quercetin demonstrates a broad mechanistic profile that aligns closely with multiple pathophysiological processes involved in major depressive disorder.

### 5.3 Resveratrol

Resveratrol is widely researched as an activator of SIRT1, which belongs to the class III histone deacetylases (HDACs) and plays critical roles in cellular functions such as stress response, apoptosis, and mitochondrial function (229). In an experiment, chronic restraint stress (CRS) led to depressive behaviors in male Wistar rats, evident from the reduced preference for sucrose and increased immobility duration. Resveratrol effectively counteracted these behavioral changes induced by CRS. The treatment also normalized reduced levels of BDNF and phosphorylated extracellular signal-regulated kinase (p-ERK) in the hippocampus and prefrontal cortex (PFC), and restored mRNA expression levels of Bcl-2 and Bax. These effects of resveratrol closely resembled those observed with fluoxetine, suggesting comparable antidepressant-like properties. Thus, resveratrol may mitigate CRS-induced depression-like behaviors in rats by influencing apoptotic mechanisms and enhancing BDNF and p-ERK signaling in specific brain regions (230). In a study, inflammation was observed to induce oxidative stress and impair mitochondrial function in the hippocampus, resulting in depressive behaviors like decreased preference for sucrose and increased immobility during the forced swimming test. Both Mito-TEMPO, a specific antioxidant targeting mitochondria, and resveratrol successfully alleviated these depressive symptoms. Conversely, blocking the mitochondrial respiratory chain with rotenone also caused depressive-like behaviors, underscoring the association between mitochondrial dysfunction and depression. Furthermore, resveratrol reduced inflammation-induced cell apoptosis in the hippocampus, demonstrating its antidepressant effects by counteracting mitochondrial oxidative stress and preventing cell death (231). Estrogen deficiency often leads to depression and anxiety, both of which are linked to inflammation. Resveratrol demonstrated effectiveness in reducing anxiety and depression-like behaviors in ovariectomized mice by mitigating inflammatory processes. The research indicated that resveratrol treatment alleviated these symptoms, reduced microglial activation in the hippocampus, and inhibited the activation of NLRP3 and NF-κB triggered by estrogen deficiency. These antidepressant effects were due to resveratrol's capacity to boost SIRT1 levels and decrease the production of inflammatory cytokines in the hippocampus (232). The study explored how resveratrol influences energy levels and neurotransmission in the hippocampus, specifically examining its antidepressant effects. The findings indicated that resveratrol, when combined with fluoxetine, successfully alleviated depressive-like behaviors triggered by CUMS. Resveratrol increased ATP production, lowered Na+-K+-ATPase and pyruvate levels, and stimulated mitochondrial DNA (mtDNA) and important gene expression linked to mitochondrial function. Additionally, it elevated 5-HT levels and decreased the serotonin transporter (SERT) expression, thereby improving neurotransmission. Moreover, it counteracted the decrease in growth-associated protein (GAP)-43 caused by CUMS, underscoring its ability to enhance neural plasticity (233).

Resveratrol engages several of the major biological pathways implicated in depressive disorders, providing a mechanistic rationale for its antidepressant potential. One of its most consistent actions is the suppression of microglial activation and the inhibition of inflammatory signaling cascades. Resveratrol downregulates NF-κB activity and decreases the expression of NLRP3 inflammasome components, thereby reducing the production of proinflammatory cytokines that contribute to neuronal dysfunction and behavioral symptoms of depression. By limiting neuroinflammation at multiple regulatory points, resveratrol acts directly on pathways known to influence mood, cognition, and stress responsivity (232, 234).

In parallel, resveratrol demonstrates robust effects on mitochondrial function and oxidative balance. Chronic stress models consistently show that resveratrol restores ATP production, stabilizes mitochondrial membrane potential, and reduces excessive generation of ROS and NOS. These protective effects counteract mitochondrial impairment and oxidative damage that are frequently observed in depressive states. Through this mitochondrial support, resveratrol improves cellular energy metabolism and reduces molecular injury that would otherwise exacerbate depressive behaviors (231, 235).

Resveratrol also influences monoaminergic regulation. Experimental evidence indicates that it enhances the availability of serotonin by reducing the expression of the serotonin transporter, thereby increasing synaptic 5-hydroxytryptamine levels. This effect on neurotransmission complements its anti-inflammatory and antioxidant properties, contributing to the overall normalization of behavioral outcomes in stress-induced models of depression (236, 237). Additionally, resveratrol promotes neuroplasticity by reversing stress-related reductions in BDNF and increasing the expression of proteins associated with synaptic remodeling (230). Through these combined actions on inflammatory signaling, mitochondrial function, oxidative stress, neurotransmitter balance, and neuroplasticity, resveratrol engages a broad network of depression-relevant pathways and provides strong preclinical support for its potential use as a therapeutic agent.

### 5.4 Catechins

EGCG, the most prevalent catechin in green tea, is recognized as a powerful antioxidant. Due to its diverse effects, including anti-inflammatory, anti-apoptotic, and autophagy-promoting properties, it has been extensively used for preventing and treating various diseases (238). The potential of EGCG as an antidepressant was investigated in mice subjected to prolonged stress. EGCG mitigated depression-like behaviors observed in the FST. It decreased IL-1β levels in the bloodstream, boosted BDNF mRNA levels in the hippocampus, and shielded CA3 neurons from structural damage. These outcomes indicate that EGCG may alleviate depression by regulating inflammation, enhancing BDNF production, and safeguarding hippocampal neurons (239). Epicatechin (Epi), a flavonoid abundantly present in cocoa, has been demonstrated to alleviate anxiety and depressive symptoms in mice. This research explored the impact of Epi on depression-like behaviors caused by chronic mild stress (CMS) in male mice. Epi was given orally twice daily over a period of five weeks. Results from behavioral assessments, including the sucrose preference and open field tests, showed that Epi reduced anhedonia and anxiety-like behavior. The antidepressant properties of Epi were linked to its regulatory effect on the aminotransferase enzyme KAT, indicating that Epi bolstered resilience against stress-induced depression (240). In a study, mice exposed to CUMS exhibited depression-like behaviors, which were mitigated by EGCG treatment. The improvement in behavior was indicated by higher sucrose preference and shorter immobility time in FST. EGCG also diminished the activation of the NLRP3 inflammasome and its downstream inflammatory mediators, including IL-1β and IL-18. Moreover, EGCG suppressed the mTOR signaling pathway and restored autophagy by regulating markers such as Beclin-1, LC3, and p62, while also reducing the expression of apoptosis markers Bax and Bcl-2. These results indicate that the antidepressant effects of EGCG are achieved through various mechanisms, including anti-inflammatory actions, autophagy regulation, and inhibition of apoptosis (241).

Catechins exert antidepressant-like effects through a wide range of biological pathways that correspond closely with the major mechanisms implicated in depressive disorders. Beyond their well-established antioxidant capacity, catechins such as EGCG directly modulate neuroinflammatory processes by reducing the activation of key inflammatory complexes, including the NLRP3 inflammasome. This suppression of inflammatory signaling decreases the production of cytokines that impair neuronal function and contribute to depressive behaviors (241, 242). Catechins also restore autophagy balance, preventing the accumulation of damaged cellular components that can trigger neurodegeneration and exacerbate mood disturbances (243). In parallel, catechins consistently demonstrate protective effects on hippocampal structure and function. Both EGCG and epicatechin promote the survival of pyramidal neurons and prevent stress-induced morphological damage, which is essential for maintaining healthy emotional regulation (240, 244). These compounds also enhance the expression of BDNF and other synaptic proteins involved in learning, memory, and mood stabilization. By supporting neuroplasticity, catechins counteract one of the hallmark deficits observed in depressive states (245, 246).

Furthermore, catechins influence neurotransmission and metabolic activity in ways that complement their anti-inflammatory and neuroprotective actions. Epicatechin has been shown to modulate enzymes such as kynurenine aminotransferase, which plays an important role in stress resilience and glutamatergic regulation. These effects help stabilize neural circuits that become dysregulated during chronic stress (247). Finally, catechins have meaningful interactions with the gut–brain axis. Studies demonstrate that these compounds help maintain a healthier balance of gut microbiota, prevent stress-related reductions in beneficial bacterial species, and preserve short-chain fatty acid production. Because disturbances in microbiota composition and microbial metabolites are increasingly recognized as contributors to depressive symptoms, this interaction provides an additional mechanistic link connecting catechins to core biological pathways associated with major depressive disorder (247–249). Through their simultaneous actions on inflammation, oxidative stress, neuroplasticity, neurotransmitter function, and gut–brain communication, catechins influence multiple interconnected mechanisms that are central to the development and persistence of depression. These combined effects offer strong preclinical support for their potential role as therapeutic agents.

### 5.5 Ferulic acid

Ferulic acid deserves independent discussion rather than being grouped under miscellaneous polyphenols. Beyond being a widely distributed hydroxycinnamic acid, ferulic acid holds particular pharmacognostic significance because it is used as a core indicator component in the Chinese Pharmacopeia for evaluating the quality of medicinal and edible homologous herbs such as *Angelica sinensis* and *Ligusticum chuanxiong*. This position gives ferulic acid a distinctive relevance at the intersection of traditional Chinese medicine, functional foods, and modern antidepressant research.

Experimental evidence consistently supports the antidepressant potential of ferulic acid in several animal models. In chronic unpredictable mild stress, ferulic acid significantly improved sucrose preference and reduced behavioral despair, while simultaneously increasing BDNF, PSD95, and synapsin I levels in both the hippocampus and prefrontal cortex, indicating enhancement of neurotrophic support and synaptic plasticity (250). In chronic corticosterone exposed mice, ferulic acid reversed depressive-like behavior and reduced oxidative stress, suggesting a protective effect against HPA axis related pathological changes (251). Similarly, in reserpine treated mice, ferulic acid ameliorated both depression-like behavior and pain related abnormalities, restored serotonin, norepinephrine, and dopamine levels, and attenuated oxidative, inflammatory, and apoptotic signaling in the frontal cortex and hippocampus (252).

The mechanistic profile of ferulic acid is notably broad. Earlier work showed that ferulic acid exerted antidepressant and prokinetic effects similar to Chaihu Shugan San, acting through regulation of monoaminergic systems, HPA axis mediators, and ghrelin signaling, thereby illustrating a clear polypharmacological pattern rather than a single target effect (253). More recent work has further emphasized its anti inflammatory value in complex disease contexts. In epilepsy associated depression, ferulic acid supplementation restored circulating corticosterone, reduced IL-1β, TNF-α, and indoleamine 2,3 dioxygenase activity, and improved depression-related outcomes when combined with levetiracetam (254). A recent review also summarized the holistic antidepressant mechanisms of ferulic acid, including regulation of monoamine and non-monoamine neurotransmitters, attenuation of neuroinflammation and oxidative stress, promotion of hippocampal neurogenesis, upregulation of BDNF, and protection against mitochondrial dysfunction and apoptosis (255). Taken together, these findings indicate that ferulic acid is not a minor adjunctive compound, but rather one of the most mechanistically versatile phenolic acids in the antidepressant polyphenol field.

Its relevance is further strengthened by the broader antidepressant evidence surrounding *Angelica sinensis* and *Ligusticum chuanxiong*, two traditional Chinese medicinal herbs in which ferulic acid is a representative quality marker. Studies on *Angelica sinensis* extracts have shown antidepressant effects in chronic stress models through upregulation of BDNF, CREB, and ERK signaling in the hippocampus (256), as well as through regulation of neuroendocrine immune imbalance and sphingolipid metabolism (257). Additional studies reported that *Angelica sinensis* improved depressive symptoms together with hematological and metabolic abnormalities in stress induced depression (258). Likewise, herb pair studies involving *Angelica sinensis* and *Ligusticum chuanxiong* demonstrated antidepressant effects associated with increased monoamines, reduced inflammatory cytokines, improved blood rheology, and activation of PI3K/AKT and BDNF related signaling (259). Although these herbal effects cannot be attributed exclusively to ferulic acid, they reinforce the academic and translational importance of ferulic acid as a core bioactive and quality control constituent in this pharmacological tradition.

Collectively, the evidence on this polyphenolic compound demonstrates that they exert antidepressant actions through several interconnected biological pathways that are central to depression. Ferulic acid consistently enhances neuroplasticity by increasing the expression of BDNF, synaptic proteins, and regulators of neuronal connectivity in the hippocampus and prefrontal cortex. This improvement in neurotrophic signaling supports neuronal resilience and restores behavioral function under chronic stress (250, 260). Ferulic acid also reduces oxidative stress and restores antioxidant capacity by lowering lipid peroxidation and enhancing endogenous antioxidant systems, thereby protecting neural tissue from stress-related damage. These antioxidant and neurotrophic effects work in parallel with its ability to modulate the hypothalamic–pituitary–adrenal axis and normalize corticosterone levels, which contributes to improved regulation of the stress response (251, 261).

### 5.6 Luteolin

Luteolin has emerged as an important flavone in antidepressant research, with evidence spanning stress related, inflammatory, neurodegenerative, and late onset depression models. Its growing relevance lies in the fact that its actions extend beyond antioxidant defense and include regulation of HPA axis activity, neurotransmitter balance, neuroinflammation, synaptic plasticity, endoplasmic reticulum stress, and cellular fate programs.

In a post traumatic stress disorder model induced by single prolonged stress, luteolin reduced fear, anxiety, and depression-like behavior while suppressing elevations in corticosterone and adrenocorticotropic hormone. It also corrected monoaminergic imbalance by normalizing norepinephrine and serotonin levels in the medial prefrontal cortex and hippocampus, suggesting that its behavioral effects are linked to both HPA axis stabilization and neurotransmitter homeostasis (262). Similarly, in noise induced depression, luteolin significantly ameliorated behavioral deficits and suppressed neuroinflammation in the hippocampus and prefrontal cortex, while also increasing synapsin expression and restoring serum serotonin and norepinephrine levels (263). These observations indicate that luteolin can preserve both biochemical and synaptic aspects of mood regulation under chronic environmental stress.

More recent studies have expanded the mechanistic profile of luteolin. In late onset depression rats, luteolin improved depressive and anxiety-like behaviors as well as cognitive impairment, and metabolomic analysis indicated that these effects were associated with modulation of glycerophospholipid metabolism in the hippocampus and prefrontal cortex. Interestingly, the authors further linked these regional lipid changes to bidirectional regulation of autophagy, suggesting that luteolin may act through metabolic remodeling rather than only through classical inflammatory pathways (264). In an Alzheimer related model, luteolin also alleviated depression-like behavior by suppressing endoplasmic reticulum stress, reducing IL-1β production, and attenuating microglial activation, thus connecting its antidepressant action to ER stress and innate immune signaling (265).

Additional evidence suggests that luteolin may influence developmental and neurotrophic processes relevant to depression. In human neural stem cells and in an LPS induced depression model, luteolin modulated signaling pathways related to neurotrophins, Wnt, BMP, JAK-STAT, dopamine, and inflammation. It also reduced IL-6, TNF-α, and corticosterone while increasing mature BDNF, dopamine, and noradrenaline levels, supporting a broader role in neural plasticity and neuroimmune regulation (266). Thus, these data indicate that luteolin is no longer merely an auxiliary flavonoid in antidepressant research, but a mechanistically rich candidate with effects spanning stress endocrinology, monoamines, synaptic function, neuroinflammation, and metabolic homeostasis.

### 5.7 Apigenin

Apigenin also deserves a dedicated section because its antidepressant related effects have been repeatedly demonstrated across inflammatory, stress induced, endocrine, and oxidative injury based models. Compared with many other polyphenols, apigenin stands out for the consistency with which it modulates inflammatory signaling, neurotrophic pathways, oxidative stress, and neuronal survival.

In an LPS induced model, apigenin prevented increased immobility and reduced sucrose preference, while significantly suppressing IL-1β, TNF-α, iNOS, and COX-2 expression in the prefrontal cortex through inhibition of NF-κB activation (267). In chronic mild stress, its mechanism was refined further, as apigenin was shown to ameliorate depressive behavior by upregulating PPARγ and inhibiting NLRP3 inflammasome activation and IL-1β production in the rat brain (268). These findings are especially important because they place apigenin among the relatively few polyphenols for which inflammasome related mechanisms have been explicitly demonstrated in depression models.

Apigenin also exerts significant antioxidant and metabolic regulatory effects. In a streptozotocin mediated depression model, it improved behavioral outcomes, restored antioxidant defenses, elevated coenzyme Q10, and downregulated Nlrp3, Tlr4, and AMPK associated abnormalities, suggesting an integrated action on oxidative stress, inflammatory signaling, and cellular energy homeostasis (269). In chronic mild stress mice, apigenin reduced immobility, increased sucrose preference, lowered corticosterone and nitrite, and improved glutathione and lipid peroxidation related parameters, further supporting its antioxidant and endocrine regulatory actions (270).

Neurotrophic and anti apoptotic mechanisms also appear to contribute to the antidepressant profile of apigenin. In corticosterone induced depression, apigenin reversed behavioral abnormalities while restoring hippocampal BDNF levels and reducing serum corticosterone, indicating involvement of neurotrophic signaling and HPA axis normalization (271). More recently, apigenin was shown to increase serotonin, dopamine, and norepinephrine, enhance CREB and BDNF protein expression in the hippocampus, reduce neuronal apoptosis, and inhibit pro inflammatory microglial activation in both *in vivo* and *in vitro* models (272). Apigenin demonstrates a broad spectrum of activity that includes antioxidant protection, suppression of inflammatory cytokines, and inhibition of pathways such as TLR4 and NLRP3 that are involved in neuroimmune activation. Through these mechanisms, apigenin reduces neuronal injury, improves energy homeostasis, and restores behavioral parameters impaired by chronic stress. It also modulates monoamine oxidase A activity, further contributing to improvements in neurotransmission (268, 269, 273, 274).

Altogether, the available evidence indicates that apigenin acts through a genuinely multi target mechanism involving inflammation, oxidative stress, inflammasome activity, neurotrophic support, monoamines, and cell survival pathways.

### 5.8 Other emerging polyphenolic compounds

In addition to the major compounds discussed above, several other polyphenols have shown antidepressant related promise and should be acknowledged to reflect the breadth of the field. p Coumaric acid, for example, has demonstrated significant antidepressant effects in chronic restraint stress models. It appears to act as a 5-HT6 receptor antagonist and has been reported to reduce immobility, lower CORT, CRH, and ACTH levels, increase serotonin, dopamine, and norepinephrine, reduce MAO-A and SERT expression, and activate ERK, CaMKII, and Akt/mTOR related pathways in brain regions associated with mood regulation (275). These findings suggest that p coumaric acid may combine monoaminergic, neuroendocrine, and intracellular signaling effects in a manner comparable to more established antidepressant polyphenols.

p-Coumaric acid influences additional pathways involved in depressive behaviors. It increases monoaminergic neurotransmission by raising levels of serotonin, dopamine, and noradrenaline in key brain regions while simultaneously reducing monoamine oxidase activity and modulating serotonin transporter expression. These actions help restore neurotransmitter balance and support behavioral recovery (275). p-Coumaric acid also reduces behavioral and endocrine consequences of stress by lowering corticotropin-releasing hormone and adrenocorticotropic hormone, indicating a regulatory effect on stress-related endocrine signaling (276). Its influence on intracellular cascades including ERK, CaMKII, and Akt pathways further supports neuroplasticity and synaptic function (277, 278).

Genistein is another example of an emerging compound with mechanistic interest. This soybean derived isoflavone crosses the blood brain barrier and has demonstrated antidepressant effects associated with increased monoamine levels and suppression of monoamine oxidase activity. Its effects were attenuated by serotonin depletion and modulated by 5-HT1A receptor interventions, indicating that serotonergic signaling plays a central role in its antidepressant action (279). This receptor linked mechanism gives genistein a distinct pharmacological profile compared with compounds that act more prominently through oxidative stress or inflammatory pathways. Genistein also enhances monoaminergic signaling, inhibits monoamine oxidase, and exerts its antidepressant effects through serotonergic mechanisms, particularly the 5-HT1A, which plays a central role in mood regulation. Genistein also provides antioxidant and anti-inflammatory benefits, contributing to improved neuronal health and reduced vulnerability to stress (280).

The broader traditional medicine literature also suggests that additional polyphenol rich herbal systems may contribute valuable leads for antidepressant discovery. For instance, Danggui-Shaoyao-San improved depressive behavior in both forced swim and chronic stress paradigms and influenced the central arginine vasopressin system (281). More recent herbal pair and formula studies involving *Ligusticum chuanxiong* have identified multiple candidate antidepressant constituents and pathways, including glucocorticoid receptor related protection, PI3K/AKT signaling, neuroinflammation control, and mitophagy regulation through SIRT3/PINK1/Parkin signaling (282, 283). Although these studies investigate multi component systems rather than isolated dietary polyphenols, they emphasize that the current antidepressant polyphenol landscape is wider than a few canonical molecules and continues to expand through integration of nutrition science, pharmacognosy, and ethnopharmacology. Overall, extending the discussion beyond curcumin, quercetin, resveratrol, and catechins reveals a more diverse and mechanistically nuanced field. Ferulic acid, luteolin, and apigenin clearly deserve dedicated attention, while compounds such as p coumaric acid and genistein, together with polyphenol rich traditional herbal systems, indicate that the repertoire of potentially relevant antidepressant polyphenols remains far from exhausted.

## 6 Clinical evidence and trials

Curcumin displayed promising antidepressant effects in a six-week clinical trial involving 60 patients diagnosed with MDD, who were randomly assigned to receive fluoxetine (20 mg), curcumin (1,000 mg), or a combination of both treatments. Evaluation based on the Hamilton Depression Rating Scale, 17-item version (HAM-D17), indicated higher response rates in the group receiving the combination therapy compared to those receiving fluoxetine or curcumin alone, although these differences did not reach statistical significance. Notably, curcumin was well-tolerated with minimal adverse effects. These findings suggest that curcumin may represent a viable and safe therapeutic option for managing MDD, prompting further exploration of its mechanisms and potential synergies with conventional treatments (284) (Table 2). In a controlled trial where participants were randomly assigned to receive either curcumin or a placebo for 8 weeks, the effects of curcumin from turmeric on MDD were examined. Curcumin was administered at a dosage of 500 mg twice daily. The study utilized the Inventory of Depressive Symptomatology self-rated version (IDS-SR30) and the Spielberger State-Trait Anxiety Inventory (STAI) to assess outcomes. While both groups initially showed improvement from baseline to week 4, curcumin demonstrated notably superior efficacy compared to placebo from weeks 4 to 8 in reducing overall depressive symptoms and mood-related scores. These findings suggest that curcumin could be beneficial in treating MDD, especially in individuals with atypical depression (285). In a recent study that was randomized, double-blind, and placebo-controlled, a proprietary curcumin extract (500 mg twice daily) demonstrated partial effectiveness in reducing depressive symptoms over an 8-week period in individuals diagnosed with MDD. A secondary examination of biomarkers collected from 50 participants indicated that curcumin supplementation correlated with elevated levels of urinary thromboxane B2 and substance P, whereas placebo intake correlated with decreased levels of aldosterone and cortisol. Participants who exhibited higher baseline plasma levels of endothelin-1 and leptin demonstrated more pronounced improvements in depressive symptoms with curcumin treatment. These findings imply that curcumin may influence biomarkers associated with its potential mechanisms for treating depression, particularly plasma levels of leptin and endothelin-1 (286). In a clinical trial exploring the use of curcuminoids as an add-on to conventional antidepressants for treating MDD, participants were divided into two groups: one receiving standard antidepressant therapy along with a combination of curcuminoids and piperine and the other receiving standard antidepressant therapy alone over a period of 6 weeks. Evaluations using the Hospital Anxiety and Depression Scale (HADS) and Beck Depression Inventory II (BDI-II) revealed significant decreases in total scores and subscales related to anxiety and depression in the curcuminoids group compared to the control group. Likewise, reductions in the BDI-II total score and its somatic and cognitive subscales were notably more pronounced in the curcuminoids group. These results suggest that combining curcuminoids with piperine is a safe and effective enhancement to standard antidepressant therapy for MDD (287). Curcumin demonstrated substantial antidepressant effects in individuals diagnosed with MDD, significantly reducing both HAM-D17 and Montgomery-Asberg Depression Rating Scale (MADRS). This extended treatment regimen also induced significant alterations in inflammatory cytokines, notably decreasing levels of IL-1β and TNF-α, and concurrently increasing BDNF levels, while reducing salivary cortisol concentrations compared to those receiving a placebo. These outcomes underscore curcumin's capacity to enhance the efficacy of antidepressant treatments and potentially alleviate the progression of depression (288). Curcumin, recognized for its ability to reduce inflammation and oxidative stress, exhibited notable antidepressant effects in individuals diagnosed with MDD. A 12-week clinical trial involving 65 participants, conducted in a double-blind, placebo-controlled manner, revealed that adding curcumin to treatment regimens (dosages ranging from 500 to 1,500 mg/day) led to significant improvements in MADRS scores compared to placebo. These benefits became statistically significant by weeks 12 and 16 of the study. The antidepressant effects of curcumin were more prominent in male participants than in females. Moreover, curcumin was well-tolerated with no noteworthy adverse effects observed in blood chemistry or ECG readings. Future research should focus on longer treatment durations and higher dosages to further explore curcumin's potential as an antidepressant in MDD, particularly in combination with standard antidepressant therapies (289).

**Table 2:** Overview of clinical studies on polyphenols for MDD treatment.

Study design	Participants (*N*, diagnosis)	Intervention (polyphenol, dose, control)	Duration	Primary assessment tool	Main results	Safety notes	Ref.
Randomized, observer-masked trial	*N* = 60, MDD (DSM criteria)	Curcumin (1,000 mg/day), fluoxetine (20 mg/day), or combination	6 weeks	HAM-D17	Curcumin showed comparable efficacy to fluoxetine; combination group showed higher response rate (77.8%) but not statistically significant	Well tolerated; no major adverse effects reported	Sanmukhani et al. (284)
Randomized controlled trial (adjunctive)	*N* = 111, MDD	Curcuminoids + piperine (1,000 mg + 10 mg/day) + standard antidepressant vs. antidepressant alone	6 weeks	BDI-II, HADS	Significant improvement in depression and anxiety scores in curcuminoid group (*p* < 0.001)	Safe and well tolerated	Panahi et al. (287)
Randomized, double-blind, placebo-controlled trial	*N* = 65, MDD	Curcumin (500–1,500 mg/day) + antidepressant vs. placebo + antidepressant	12 weeks (+4-week follow-up)	MADRS, HAM-A	Significant improvement in depressive symptoms (MADRS) at weeks 12 and 16; stronger effects in males	No significant adverse effects; no changes in ECG or blood chemistry	Kanchanatawan et al. (289)
Randomized, double-blind, placebo-controlled trial	*N* = 56, MDD	Curcumin (500 mg twice daily) vs. placebo	8 weeks	IDS-SR30, STAI	Significant improvement in depressive symptoms from weeks 4–8; stronger effects in atypical depression subgroup	No major safety concerns reported	Lopresti et al. (285)
Randomized, double-blind, placebo-controlled (biomarker study)	*N* = 50, MDD	Curcumin (500 mg twice daily) vs. placebo	8 weeks	IDS-SR30	Reduction in depressive symptoms associated with biomarker modulation (e.g., endothelin-1, leptin)	No serious adverse effects reported	Lopresti et al. (286)
Randomized, double-blind, placebo-controlled trial	*N* = 123, MDD	Low-dose curcumin (250 mg b.i.d.), high-dose (500 mg b.i.d.), or curcumin + saffron vs. placebo	12 weeks	IDS-SR30, STAI	Active treatments significantly improved depressive and anxiety symptoms vs. placebo (*p* = 0.031)	Well tolerated; no major adverse effects	Lopresti et al. (290)
Observational (cross-sectional)	*N* = 93 (MDD and non-depressed)	Dietary anthocyanin intake (habitual intake assessment)	Not applicable	DASS-21	Higher anthocyanin intake associated with lower depression scores (*p* = 0.007)	No safety concerns (dietary study)	Mestrom et al. (291)

In a 12-week clinical trial involving 123 individuals diagnosed with MDD, researchers examined the therapeutic potential of curcumin and saffron in alleviating symptoms of depression and anxiety. The study, which employed a randomized, double-blind, placebo-controlled design, assigned participants to receive either placebo, low-dose curcumin (250 mg twice daily), high-dose curcumin (500 mg twice daily), or a combination of low-dose curcumin and saffron (15 mg twice daily). The findings indicated that both curcumins alone and the curcumin/saffron combination significantly mitigated depressive symptoms compared to placebo. Moreover, these treatments demonstrated enhanced efficacy in individuals with atypical depression, yielding higher response rates relative to other depression subtypes. Overall, the study underscores the potential of curcumin and saffron, whether used independently or together, as effective therapies for managing depression and anxiety symptoms in individuals diagnosed with MDD (290).

A study by Mestrom and colleagues examined habitual dietary flavonoid intake in adults with and without major depressive disorder and explored its relationship with depressive symptom severity. Using diet history interviews and flavonoid quantification based on the PhenolExplorer database, the authors found that total flavonoid intake and most flavonoid subclasses were broadly similar between groups; however, anthocyanin intake was significantly lower in participants with MDD than in non-depressed controls. Importantly, higher anthocyanin intake was associated with lower depression scores across the total sample, suggesting a possible relationship between anthocyanin-rich dietary patterns and reduced depressive symptomatology. A later full journal publication reported consistent findings, again showing lower anthocyanin intake in participants with MDD and an inverse association between anthocyanin intake and DASS-depression scores. Together, these findings highlight anthocyanins as a potentially relevant dietary subclass of flavonoids in depression research, although larger and longitudinal studies are still needed to determine causality and clinical applicability (291, 292).

## 7 Incorporating polyphenols into clinical therapy

The potential of polyphenols as therapeutic agents for MDD has garnered increasing interest due to their diverse biological activities. Although a substantial number of animal studies have demonstrated the antidepressant-like effects of various polyphenols—including catechins, curcumin, ferulic acid, resveratrol, quercetin, EGCG, p-coumaric acid, genistein, and apigenin—there is a notable gap in clinical research directly addressing their efficacy in humans. The limited number of controlled trials RCTs that have explored the effects of polyphenols on MDD have primarily focused on curcumin and anthocyanins, both of which have shown promising results.

Curcumin, a major component of turmeric, has been highlighted in several clinical studies for its potential antidepressant effects. It operates through multiple mechanisms, including modulation of neurotransmitter levels, reduction of inflammatory and oxidative stress markers, and regulation of the HPA axis. These properties make curcumin a promising candidate for incorporation into clinical therapy for MDD. Similarly, anthocyanins, which are abundant in purple and red fruits and vegetables, have demonstrated potential in reducing depressive symptoms. Clinical studies suggest that individuals with MDD tend to have a lower dietary intake of anthocyanins, and higher consumption of these compounds correlates with lower depression scores.

Given the potential benefits of polyphenols in managing depressive symptoms, integrating these compounds into the diet could be a practical and non-invasive strategy. Encouraging the consumption of polyphenol-rich foods, such as berries (rich in anthocyanins), apples, onions, turmeric (rich in curcumin), green tea, and dark chocolate, can naturally boost polyphenol intake. For those unable to consume sufficient quantities of polyphenol-rich foods, dietary supplements offer a viable alternative. Curcumin supplements, often combined with piperine to enhance bioavailability, and anthocyanin supplements derived from berries are readily available. Additionally, the development of functional foods and beverages fortified with polyphenols provides another avenue for increasing intake without significant dietary changes.

Animal studies on polyphenols like catechins, ferulic acid, resveratrol, quercetin, EGCG, p-coumaric acid, genistein, and apigenin have shown antidepressant-like effects, suggesting that these compounds may also play a role in treating MDD. Catechins and EGCG, found in green tea, have been noted for their neuroprotective and anti-inflammatory properties. Resveratrol, present in grapes and red wine, has demonstrated potential in modulating the HPA axis and reducing oxidative stress. Quercetin, a flavonoid found in many fruits and vegetables, exhibits anti-inflammatory and neuroprotective effects. Ferulic acid and p-coumaric acid, commonly found in cereals and coffee, are known for their antioxidant properties, while genistein and apigenin, found in soy products and certain herbs, respectively, have shown potential in modulating neurotransmitter levels and reducing neuroinflammation. The integration of polyphenols into conventional therapeutic regimens for MDD may offer synergistic benefits. When used alongside existing treatments, polyphenols can enhance the overall efficacy and potentially reduce the dosage and side effects of pharmaceutical antidepressants. For instance, curcumin has been shown to enhance the effectiveness of standard antidepressants and accelerate symptom reduction. Incorporating polyphenol-rich foods into the diet can serve as a preventive strategy for individuals at risk of developing MDD, leveraging the antioxidant and anti-inflammatory properties of these compounds to mitigate the onset of depressive symptoms. Hence, while the current body of evidence supports the potential role of polyphenols, particularly curcumin and anthocyanins, in managing MDD, further research is necessary to establish definitive guidelines and optimize their use in clinical settings. By exploring dietary strategies and potential synergies with existing treatments, polyphenols can be effectively integrated into comprehensive therapeutic regimens for MDD. Additionally, expanding clinical research to include other polyphenols that have shown promise in animal studies could broaden the therapeutic options available for individuals suffering from MDD.

## 8 Safety, toxicological considerations, and interaction risks of polyphenols

Although polyphenols are widely regarded as beneficial dietary constituents, their safety profile in therapeutic or pharmacological settings requires more careful consideration than is often assumed. The perception that polyphenols are intrinsically safe because they are naturally present in foods may be misleading when these compounds are administered at high doses, in concentrated extracts, through enhanced delivery systems, or over prolonged periods. This distinction is particularly important in the context of depression, where long-term treatment is often required and where patients may simultaneously receive antidepressants, anxiolytics, mood stabilizers, antiepileptic drugs, or medications for metabolic and cardiovascular comorbidities (293–295).

Available evidence suggests that the safety of polyphenols is highly dependent on compound identity, dose, formulation, duration of exposure, and host-related factors such as age, liver function, intestinal microbiota, nutritional status, and concomitant drug use. While many polyphenols have shown favorable tolerability in short-term preclinical and clinical studies, the toxicological database remains uneven and incomplete. The use of concentrated preparations may lead to biological effects that differ substantially from those associated with habitual dietary intake. In some cases, polyphenols may show biphasic or hormetic behavior, exerting protective effects at moderate levels but potentially producing pro-oxidant, cytotoxic, endocrine, or metabolic disturbances at excessive concentrations (294–296).

Another major issue concerns long-term and high-dose exposure. Because several polyphenols have limited oral bioavailability, there is a tendency in both experimental and commercial settings to increase the administered dose or to use formulation strategies designed to boost absorption. While this may improve efficacy, it may also alter tissue distribution, systemic exposure, hepatic burden, and off-target effects. Current reviews also indicate that *in vivo* toxicity data after oral administration remain concentrated on only selected classes of extracts, such as green tea, grape-derived phenolics, and anthocyanin-rich products, which means that the evidence base is still incomplete for broad generalization across polyphenols (296–298).

Potential interactions with other medications must also be considered carefully. Polyphenols may influence drug pharmacokinetics through effects on cytochrome P450 enzymes, uridine diphosphate glucuronosyltransferases, sulfotransferases, intestinal efflux transporters, and plasma protein binding. As a result, they may increase or decrease the bioavailability of co-administered drugs. This is clinically important in patients receiving antidepressants, antipsychotics, benzodiazepines, antiepileptic drugs, anticoagulants, antihypertensives, or hypoglycemic agents. In parallel, pharmacodynamic interactions are also plausible. Polyphenols that affect monoaminergic transmission, inflammatory cascades, platelet function, glutamatergic signaling, or hypothalamic-pituitary-adrenal axis activity may amplify, attenuate, or unpredictably modify the effects of standard medications. For example, compounds with serotonergic or monoamine oxidase-modulating properties may require caution when combined with conventional antidepressants, whereas compounds with antiplatelet activity may raise concern in patients using anticoagulants or antiplatelet drugs (295, 299–301).

Safety evaluation should also extend beyond general tolerability and include organ-specific toxicity, reproductive toxicity, neurobehavioral safety, and special population risk. Patients with depression are often medically heterogeneous, and some may have impaired hepatic metabolism, chronic inflammatory states, epilepsy, cardiovascular disease, pregnancy-related vulnerability, or advanced age. In such populations, even compounds with good general safety records may behave differently. Furthermore, data derived from food consumption cannot be directly extrapolated to standardized extracts, nanoformulations, phytopharmaceutical combinations, or purified compounds administered repeatedly at supranutritional doses (293, 295, 298).

For these reasons, future translational work should integrate safety assessment as a core rather than secondary objective. Preclinical studies should include dose-ranging toxicology, repeated-dose exposure studies, pharmacokinetic profiling, organ toxicity evaluation, and interaction screening with commonly prescribed psychotropic and non-psychotropic medications. Clinical studies should systematically report adverse events, withdrawal rates, biochemical and hematological parameters, and interaction-related signals, particularly in long-term trials and adjunctive treatment settings. Establishing such evidence will be essential for distinguishing promising polyphenols with realistic clinical value from compounds whose apparent efficacy may be limited by insufficient safety characterization (296, 297, 300). Therefore, the existing literature supports the therapeutic promise of polyphenols, but clinical translation cannot rely on efficacy data alone. A balanced appraisal of both benefit and risk is necessary. Greater attention to toxicological profiling, high-dose exposure, chronic use, and medication interaction potential will strengthen the path toward safe and evidence-based integration of polyphenols into depression management.

## 9 Blood-brain barrier penetration as a translational bottleneck for polyphenols

A major challenge in translating the antidepressant potential of polyphenols into clinically meaningful central nervous system effects is their limited access to the brain. The blood-brain barrier is a highly selective interface formed by brain endothelial cells, tight junctions, transport systems, and efflux mechanisms that collectively restrict the entry of many bioactive molecules into neural tissue. Although this barrier is essential for maintaining cerebral homeostasis, it also represents one of the principal reasons why promising neuroactive compounds show weak or inconsistent efficacy *in vivo* despite strong mechanistic effects in cell-based experiments (302). This issue is particularly relevant to polyphenols because many of these compounds have poor oral bioavailability, low aqueous stability, extensive first-pass metabolism, and rapid systemic elimination. As a result, the concentration of unmetabolized parent compounds reaching the brain is often very low. Earlier work on dietary polyphenols emphasized that the journey from intestinal absorption to brain exposure is highly restrictive and that selective permeability across the blood-brain barrier, together with rapid biotransformation, may substantially limit neuroprotective efficacy if brain delivery is assumed rather than demonstrated (303).

Importantly, blood-brain barrier penetration is not uniform across polyphenols, and marked differences have been observed among both native compounds and their metabolites. Experimental permeability analyses have shown that some flavonoids and related compounds display only modest or negligible penetration, whereas others exhibit intermediate or relatively higher permeability. In one comparative study, apigenin, luteolin, quercetin, and kaempferol showed medium permeability, while epicatechin, rutin, fisetin, resveratrol, and curcumin showed negligible permeability in the applied model, underscoring that central nervous system efficacy cannot be generalized across all polyphenols as a single class (304). An important conceptual advance in this field is the growing recognition that circulating low molecular weight metabolites may be more relevant to brain exposure than the original dietary molecules themselves. Recent work demonstrated that selected sulfated low molecular weight polyphenol metabolites were capable of crossing the blood-brain barrier *in vitro* and rapidly reaching the brain *in vivo*, supporting the view that the biological effects attributed to dietary polyphenols may, at least in part, depend on their biotransformation products rather than on the parent compounds alone (305). Beyond simple penetration, recent evidence also suggests that some low molecular weight polyphenol metabolites may actively modulate blood-brain barrier biology. These metabolites have been proposed to influence barrier homeostasis by affecting oxidative stress, inflammatory tone, junctional integrity, and transporter-related processes, which raises the possibility that certain polyphenol derivatives may exert dual actions by both reaching the brain and modulating the barrier itself. This perspective is particularly relevant for depression, where neuroinflammation, endothelial dysfunction, and altered neurovascular communication may coexist with impaired neuronal signaling (306).

Because of these limitations, improving brain delivery has become a key priority for translational research. Recent reviews have highlighted liposomal and other nanocarrier based systems as promising approaches to enhance polyphenol stability, prolong circulation, improve tissue targeting, and increase effective exposure within the central nervous system. In parallel, intranasal administration has attracted attention as an alternative route capable of partially bypassing the blood-brain barrier through olfactory and trigeminal pathways, thereby offering a potentially useful strategy for selected natural products with otherwise poor brain penetration (307, 308). Thus, the limited blood-brain barrier penetration of polyphenols should be regarded as a central translational constraint rather than a secondary pharmacokinetic detail. Future studies should more consistently distinguish between parent compounds and active metabolites, directly quantify brain exposure, and incorporate delivery strategies that address the barrier problem explicitly. Such efforts will be essential for interpreting preclinical findings more accurately and for identifying which polyphenols have realistic potential for clinical application in depression.

## 10 Future directions and challenges

The therapeutic potential of polyphenols in major depressive disorder has been supported by a growing body of preclinical evidence and a limited number of clinical studies. However, successful clinical translation requires a shift from exploratory investigation toward more rigorous, standardized, and mechanistically informed research frameworks. Future directions should therefore prioritize not only efficacy, but also reproducibility, pharmacokinetic characterization, and clinically meaningful endpoints.

One of the most immediate priorities is the expansion of well-designed randomized controlled trials across a broader spectrum of polyphenols beyond curcumin and anthocyanin-rich interventions. Future trials should incorporate larger sample sizes, longer intervention durations, and stratified patient populations, including individuals with treatment-resistant depression, comorbid metabolic disorders, or inflammatory phenotypes. In addition, comparative studies evaluating whole-food interventions vs. standardized extracts or purified compounds may help clarify whether observed benefits are attributable to isolated molecules or synergistic dietary patterns.

A critical and often underappreciated challenge in this field is the lack of standardization of polyphenol interventions. Natural extracts can vary substantially in composition depending on plant source, cultivation conditions, extraction methods, and formulation. For example, products labeled as “green tea extract” may differ markedly in their epigallocatechin gallate (EGCG) content, leading to significant variability in biological exposure and clinical outcomes. This heterogeneity complicates cross-study comparisons and limits reproducibility. To address this issue, future studies should adopt chemically characterized and standardized formulations, with precise quantification of active constituents and batch-to-batch consistency.

Beyond compositional standardization, there is a strong need for biomarker-based standardization of exposure and response. Reliance on administered dose alone is insufficient, particularly given the complex metabolism and variable bioavailability of polyphenols. Future clinical trials should incorporate pharmacokinetic and metabolomic endpoints, including the measurement of circulating polyphenol metabolites (e.g., glucuronidated, sulfated, or microbiota-derived derivatives) in plasma or urine. Such biomarkers can provide a more accurate representation of systemic exposure and may help establish dose–response relationships. In parallel, the identification of biological response markers, such as changes in inflammatory cytokines, oxidative stress indices, neurotrophic factors (e.g., BDNF), or neuroimaging correlates, could support the development of mechanism-informed and personalized intervention strategies.

Another key priority is the integration of inter-individual variability into study design. Factors such as gut microbiota composition, genetic polymorphisms in metabolic enzymes, dietary background, and baseline inflammatory status may significantly influence both polyphenol metabolism and therapeutic response. Future studies should consider stratification or subgroup analyses based on these variables, as well as the incorporation of multi-omics approaches to better understand responder vs. non-responder profiles.

From a translational perspective, it is also important to establish clearer links between mechanistic findings and clinically meaningful outcomes. While many studies report modulation of neurotransmitter systems, oxidative stress, or inflammatory pathways, these effects are often not directly correlated with symptom improvement. Future research should aim to bridge this gap by integrating molecular, biochemical, and behavioral endpoints within the same study framework.

Finally, greater harmonization of study design, reporting standards, and outcome measures will be essential for advancing the field. The adoption of standardized reporting guidelines, including detailed characterization of polyphenol composition, administration protocols, and biomarker measurements, will improve comparability across studies and facilitate meta-analyses. Such efforts will be critical for determining whether polyphenols can be reliably translated into evidence-based adjunctive strategies for the management of major depressive disorder.

## 11 Conclusions

The burgeoning field of polyphenol research offers exciting prospects for advancing the treatment landscape of major depressive disorder (MDD). While animal studies have provided compelling evidence of the antidepressant potential of polyphenols such as curcumin, anthocyanins, and others, clinical validation through randomized controlled trials remains essential. Current evidence underscores the multifaceted mechanisms of action of polyphenols, including their influence on neurotransmitter pathways, inflammatory processes, oxidative stress, and neuroplasticity. However, challenges such as poor bioavailability, variability in product composition, and potential drug interactions necessitate careful consideration in future research and clinical application. Thus, there is a critical need for robust clinical trials encompassing diverse polyphenols, rigorous mechanistic investigations to elucidate pathways of action, and innovative approaches to enhance bioavailability and standardization. These efforts are pivotal in establishing polyphenols as viable adjuncts or alternatives to conventional antidepressant therapies. Public awareness and education regarding the role of polyphenol-rich diets in mental health are also imperative. By addressing these challenges and leveraging the therapeutic potential of polyphenols, we can aspire to optimize treatment outcomes and quality of life for individuals affected by MDD.
